# Directed growth and fusion of membrane-wall microdomains requires CASP-mediated inhibition and displacement of secretory foci

**DOI:** 10.1038/s41467-023-37265-7

**Published:** 2023-03-23

**Authors:** Inês Catarina Ramos Barbosa, Damien De Bellis, Isabelle Flückiger, Etienne Bellani, Mathieu Grangé-Guerment, Kian Hématy, Niko Geldner

**Affiliations:** 1 Department of Plant Molecular Biology, Faculty of Biology and Medicine, University of Lausanne, 1015 Lausanne, Switzerland; 2 Institut Jean-Pierre Bourgin, INRAe, AgroParisTech, Université Paris-Saclay, 78000 Versailles, France

**Keywords:** Plant cell biology, Plant development

## Abstract

Casparian strips (CS) are aligned bands of lignin-impregnated cell walls, building an extracellular diffusion barrier in roots. Their structure profoundly differs from tight junctions (TJ), analogous structures in animals. Nonetheless, CS membrane domain (CSD) proteins 1-5 (CASP1-5) are homologues of occludins, TJ components. CASP-marked membranes display cell wall (matrix) adhesion and membrane protein exclusion. A full CASP knock-out now reveals CASPs are not needed for localized lignification, since correctly positioned lignin microdomains still form in the mutant. Ultra-structurally, however, these microdomains are disorganized, showing excessive cell wall growth, lack of exclusion zone and matrix adhesion, and impaired exocyst dynamics. Proximity-labelling identifies a Rab-GTPase subfamily, known exocyst activators, as potential CASP-interactors and demonstrate their localization and function at the CSD. We propose that CASP microdomains displace initial secretory foci by excluding vesicle tethering factors, thereby ensuring rapid fusion of microdomains into a membrane-cell wall band that seals the extracellular space.

## Abstract (web summary)

Casparian strips are extracellular diffusion barriers in plant roots. Here, Barbosa et al. provide evidence that CASP proteins shape Casparian strips by displacing vesicle tethering factors and organizing cell wall formation

## Introduction

Plants acquired multicellularity independently of animals and thus evolved independent solutions to the many challenges that arose with increases in complexity^1^. One fundamental feature of plants is the presence of a cell wall that allows their cells to resist high internal pressure, enabling rapid cell growth through a dominating central vacuole^2^. Yet, cell walls necessarily immobilize and isolate cells, not allowing for cell-to-cell contact of plasma membranes, something which animals rely on extensively during development and cellular signalling. Thus, many of the profound differences between animal and plant development can be seen as resulting from the fact that plants have generated complex multicellularity from walled cells. A particularly straightforward difference arising from this, is the way polarized epithelia can be formed. Polarized epithelia restrict extracellular diffusion between different tissue layers by generating specific diffusion barriers. As a consequence, epithelia have to maintain two functionally distinct plasma membrane surfaces, facing the separate extracellular environments, allowing them to generate selective and vectorial transport. In animals, extracellular diffusion barriers are formed by tight and adherence junctions, polymeric assemblies of transmembrane proteins that enter in direct contact between neighboring epithelial cells, forming a network of joined rings^3^.

**Fig. 1:**
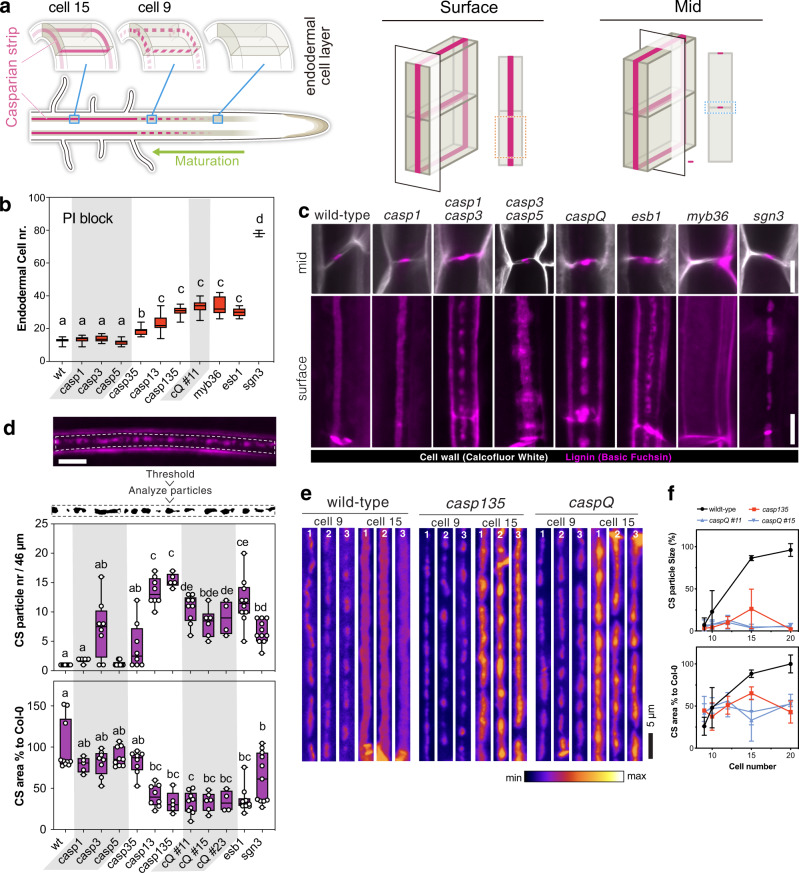
Casparian Strip development arrests at initiation sites in caspQ. **a** Schematic of Casparian strip development (magenta). Casparian strips start to appear as centrally aligned foci in the endodermal cell layer (cell 9), progressing into a network of fused rings (cell 15) (left). Illustration of lignin accumulation patterns observed in optical sections at endodermal surface (middle panel) or median positions (right panel). (Schematic modified from Fujita et al., 2019). **b** Propidium iodide uptake assay, endodermal cell number indicates position at which uptake is blocked. Box-plot with 25–75th percentiles, mean, and whiskers of minima to maxima from *n* = 10 individuals and significant differences to wild-type shown with different letters by ONE-WAY ANOVE, Tukey test comparisons with adjusted *p* value < 0.05. **c** Mid (upper panel) and surface (lower panel) views of CS-lignin (magenta, Basic Fuchsin) and cell wall (gray, Calcofluor White) staining in wild-type, CS mutants and *caspQ* at endodermal cell 20. **d** Particle analysis in CS surface of endodermal cell 20, as described in Material and Methods, with variables Particle number and CS % area to Wild-type measured per 46 µm of CS surface. Box-plot with 25–75th percentiles, mean, and whiskers of minima to maxima from *n* = 8 individuals and significant differences to wild-type shown with different letter by one-way ANOVA, Tukey test comparisons with adjusted *p* value < 0.05. **e** Representative pictures of CS surface views from three individuals at endodermal cell number 9 (start of detectable Basic Fuchsin signal) and cell 15 from wild-type, *casp135* and *caspQ*. **f** Particle size (%) and CS area (%), estimated as in (**c**), relative to mean wild-type at cell 20, shown is the mean and standard deviation from *n* = 6 individuals. Scale bars 5 µm.

Because of their cell walls, plants cannot achieve extracellular (apoplastic) diffusion barriers by membrane protein-mediated cell-to-cell contacts. Instead, a more complex interplay between transmembrane proteins and cell wall enzymes must generate a localized, coordinated membrane-wall adhesion, guiding hydrophobic cell wall modifications between cells. We are only beginning to understand the complex molecular identity of this plasma membrane - cell wall - plasma membrane nexus^4^. The CS is an illustrative example of such a tight membrane-wall barrier. Work from our lab initially established that the CS is made of lignin—a hydrophobic, polyphenolic polymer, impregnating cellulosic plant cell walls^5^. We then discovered the CASPARIAN STRIP MEMBRANE DOMAIN PROTEINs (CASPs)—the first proteins localized to the CS—and showed that they form an extensive, highly stable transmembrane platform^6^. CASPs form central, longitudinal rings in each endodermal cell, with their coordinated localization leading to a net-like appearance at a tissue-wide level that precisely coincides with the lignin impregnations of the CS themselves. In wild-type, CASP localization rapidly progresses from an initial stage of individual, centrally aligned microdomains (“string-of pearls” stage) to a completely fused band (Fig. 1a). These CASP microdomains are thought to bring together and restrict reactive oxygen species (ROS)-producing NADPH oxidases with ROS-utilizing peroxidases, being crucial for CS formation^7,8^. A number of other cell wall-localized proteins, such as ENHANCED SUBERIN 1 (ESB1) have also been identified to localize to the CS and to be involved in its formation^9,10^, indicating that a complex machinery is involved in locally synthesizing CS lignin. CASPs are small, four-transmembrane-span proteins, whose features, such as very strong endodermis-specific expression, high stability, lack of lateral mobility, strong association with cell wall containing fractions, as well as extensive cross-interactions in in vivo pull downs, all pointed to a central, structural role of these proteins in the formation of the CSD^6^. However, evidence for physical interactions between CASPs and cell wall enzymes has remained indirect. Loss of ESB1, for example, causes CASPs to form unstable microdomains that do not fuse^9^, yet there is no evidence for direct interaction of ESB1 and CASPs. Currently, only partial loss-of-functions for the CASP gene family have been reported. A *casp1 casp3* double mutant displayed a clearly interrupted CS and strong, ectopic lignification of endodermal cell corners^6,7^. This ectopic lignification is now understood not to result directly from reduced CASP function. Rather, it is due to the activation of a signal transduction pathway (the SCHENGEN (SGN) pathway), whose role is to surveil the integrity of the extracellular diffusion barrier, to boost Casparian strip formation and to initiate compensatory lignification in case of defects^11–14^. Activation of the SCHENGEN pathway in the *casp1 casp3* mutant clearly indicates that reduction-of-function of CASPs causes barrier defects. However, it was unknown whether the remaining, centrally aligned lignin foci are due to a CASP-independent localization and activity of lignin enzymes or whether they simply reflected the activity of the remaining CASPs in this mutant. Indeed, knock-out of the transcription factor MYB36 prevents expression of all CASPs and causes a complete absence of CS and their associated membrane domain^15^. Yet, lack of MYB36 activity affects expression of more than hundred genes, some of which are known to be involved in lignification, CASP stability or signal transduction, suggesting that MYB36 controls large parts of the endodermal differentiation program and that its phenotype cannot be subsumed to a specific absence of CASP function.

Finally, the strongest CASP1 delocalization phenotype was observed in a mutant of an exocyst subunit (*exo70a1/lotr2*)^16^. The exocyst is a conserved eukaryotic complex that is required for tethering late secretory vesicles to the plasma membrane, enabling their fusion. In yeast and animal systems, it has been demonstrated that Rab GTPases mediate vesicle fusion through recruitment or activation of exocyst subunits to secretory vesicles. Plants have undergone a specific, large diversification of EXO70 subunits, with 23 homologs in Arabidopsis^17^. This has led to the speculation that in plants, different EXO70 subunits are able to direct specific subsets of vesicles to distinct membrane domains. Indeed, in *exo70a1*, CASPs accumulate in small, unstable microdomains all over the endodermal surface^16^. This indicates that EXO70A1 is crucial for localization, but not for CASP1 secretion per se, which could occur through other EXO70 subunits^18^. Intriguingly, a tagged version of EXO70A1 transiently accumulate at the site of CASP microdomain formation, slightly preceding CASP1 localization itself^16^. This suggested a model whereby, in the endodermis, EXO70A1 mediates the selective, localized tethering of CASP-bearing vesicles to the median plasma membrane domain.

Our in-depth analysis of a full knock-out of CASP activities, together with our identification of RabA proteins as new players in CS formation, now allows us to draw a very different model of CASP action, in which CASPs are not needed for localization or activity of lignification enzymes. Rather, CASPs are needed to form a membrane domain of protein exclusion and cell wall attachment that rapidly suppresses further secretion to the same initial focus by evicting EXO70A1 secretion landmarks. This forces displacement of secretory foci along the median line, thus ensuring rapid fusion of foci into an uninterrupted band. Moreover, CASP action also appears to be required to ensure organized and spatially limited lignification, allowing for the formation of the idiotypic, flat lignin structure of specific width that motivated the use of the term “strip” in its original description^19^.

## Results

### Endodermal knockout of CASP function leads to abnormal lignin foci

To identify CASP function in Casparian Strip formation we aimed to isolate a full endodermal *CASP* mutant. CASPs belong to the family CASP-LIKES, with 39 members in Arabidopsis, belonging to the super family of eukaryotic MARVEL proteins^20^. CASP1 – CASP5 were categorized based on their strong, endodermis-specific expression and localization to the CSD. Since there were no mutants available for all CASP genes, we isolated a double mutant, based on two strong, exon-localized T-DNA insertion alleles, *casp3-1 casp5-1*, and targeted the remaining three CASPs by multiplexed CRISPR-Cas9 (Supplementary Fig. 1)^21^. With two gRNAs targeting each CASP1, 2 and 4, we retrieved a *casp quintuple* (*caspQ*) mutant, heteroallelic for the three genes, combining deletion and frame-shift mutation alleles, from which we isolated three homozygous allelic combinations (Supplementary Fig. 1). We then first measured extracellular (apoplastic) barrier function by measuring penetration of Propidium Iodide (PI) into the central vasculature (Fig. 1b). Using this established diffusion barrier assay^22^, we found *caspQ* plants had a delay in barrier formation typical for mutants with interrupted (*casp1-1 casp3-1* and *esb1-1*) or even absent CS (*myb36*). This is due to the SGN3 (GSO1)-dependent compensatory response^14^. Consequently, *sgn3* mutants, which have a defective barrier throughout the whole root, were used as a control (Fig. 1b). Next, we used Basic Fuchsin as a fluorescent lignin stain (Fig. 1c). Surprisingly, using this stain, the novel *caspQ* displayed rather similar phenotypes to the previously described *casp1 casp3* double mutant, i.e., interrupted lignin foci and the typical compensatory ectopic cell corner lignification^4,6^. Quantitative image analysis nonetheless revealed differences between *casp* mutants (Fig. 1d). Wild-type CS displayed one particle per region of interest at maturity (20 cells after onset of elongation), indicating complete fusion. By contrast, *casp* double and triple mutants, as well as *esb1*, displayed higher number of particles, while *caspQ* had less particles, but a similarly reduced total area. Thus, *caspQ* has less, but bigger lignin foci than lower order mutants (Fig. 1c, d). In wild-type, the forming CS is deposited initially in centrally aligned dots, coined “string-of-pearls” stage, that rapidly progress into a fused band (Fig. 1a). By contrast, a developmental progression analysis in selected genotypes (Wild-type, *casp1 casp3 casp5* and *caspQ)* (Fig. 1e, f) showed that both triple *casp1 casp3 casp5* and *caspQ* mutants fail to progress, with lignin foci covering only about 50% of the area at maturity, relative to wild-type, despite strong variation (Fig. 1f). This analysis supports the idea that CS development in *casp* mutants cannot progress beyond the string-of-pearls stage.

These phenotypes of the *caspQ* mutants forced us to re-consider our long-standing notion that the remaining, centrally-aligned lignin foci observed in *casp* multiple mutants are due to the remaining activity of CASP homologs. Yet, since CASP1-5 belong to the multi-gene CASP-LIKE family of 39 members, we wanted to ascertain that none of these CASPLs could somehow compensate for the loss of CASPs in *caspQ* endodermis—either by normal, basal expression or through induced expression in the mutant—and thus be responsible for the remaining, centrally aligned lignin dots. For this, we inspected expression of the CASPL-family in several RNA-Seq datasets: (i) endodermis-specific protoplast-sorted RNA-Seq data in wild-type and *myb36* (“myb36”)^23^ and (ii) RNA-Seq of CIF2 treatment, mimicking CS defects^13^. We found that seven other members of the CASPL1 clade, to which CASP1-5 belong, namely CASPL1A1, 1B1, 1B2, 1C1, 1C2, 1D1, 1D2, were either up-regulated in *myb36* mutant or in CIF2 treatment (Supplementary Fig. 3). Indeed, some of these members were already reported to be expressed in suberizing endodermal cells^24^. However, expression analysis, using fluorescent CASPL fusion proteins in both wild-type and *caspQ* mutant background revealed either no endodermal expression (1D1) or an onset of expression too late to be responsible for the initiation of the lignin microdomains seen in *caspQ* mutants (Supplementary Fig. 3). Yet, to completely rule out the contribution of these genes for CS formation, we knocked-out the six endodermis-expressed members, (CASPL1A1, 1B1, 1B2, 1C1, 1C2, 1D2) in the *caspQ* generating the *undecuple* mutant, *caspQ 6x-caspl*. Yet, even in this mutant, we could not observe any increased phenotypic severity compared to *caspQ* (Supplementary Fig. 3). We therefore concluded that *caspQ* is indeed a full *CASP* knockout of wild-type and that localization and initiation of lignin deposition in centrally aligned dots is independent of CASP activity.

### The centrally aligned dots in caspQ are foci of secretion and lignification

**Fig. 2:**
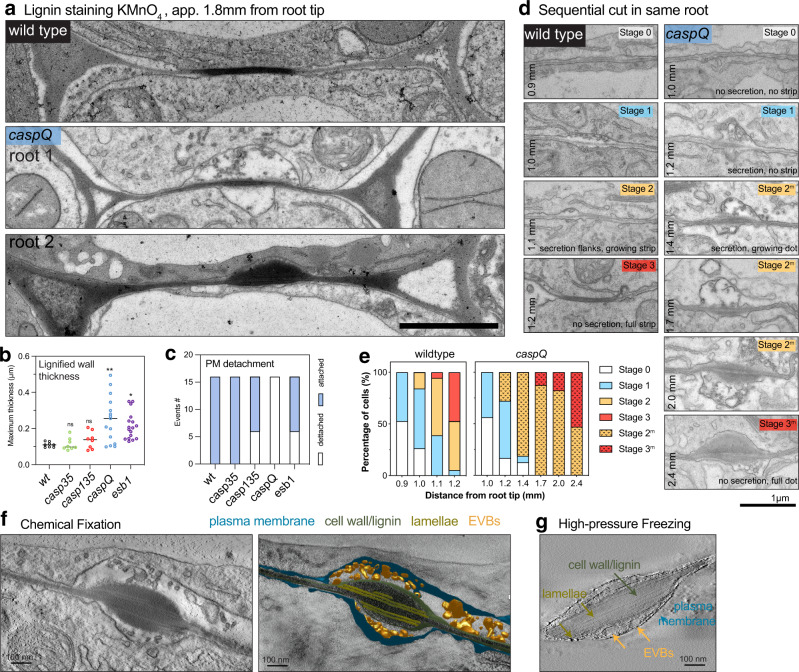
caspQ foci are lignin-rich secretion hotspots. **a–c** Representative electron micrographs with KMnO_4_ lignin staining from wild-type and *caspQ* endodermal cell-cell junctions in transversal cuts at 1.8 mm from root tip. For *caspQ* two distinctive appearances are shown. Scale bar 1 µm. **a** Quantification of maximum thickness of lignified cell-wall stained by KmnO_4_, mean (line, *n* = 16) and significant differences to wild-type by one-way ANOVA, Tukey test comparisons with adjusted *p* value < 0.05. **b** Total number of events of plasma membrane detachment in total 16 cells from two individuals (**c**). **d**, **e** Developmental staging of wild-type CS and *caspQ* foci. Pictures were taken along one root at described positions, with 8–10 endodermal cell-cell contacts, shown are pictures of one cell-cell contact for each position representative of app. 16 cells from two roots per genotype. Scale bar 1 µm. **d** *Stage 0* no detectable plasma membrane detachment, extracellular vesicles or cell-wall thickening; *Stage 1* plasma membrane detached, extracellular vesicle bodies (Evs) and beginning of cell-wall thickening; *Stage 2* wild-type CS widening: secretion stops and PM is attached to modifed cell-wall in the centre; laterally, the PM is detached and Evs are present; *Mutant stage 2 (2* ^*m*^*) caspQ* foci grow: secretion continues in centre, cell-wall thickens inwards, no PM attachment detectable. *Stage 3* mature wild-type CS: no detectable secretion, PM is attached to cell wall and CS is thin and wide. *Mutant stage 3 (3* ^*m*^*)* mature *caspQ* foci: secretion stops, cell wall is very thick but PM remains detached. Quantification of all stages from two roots per genotype, 8–10 cell-cell contacts per root **€**. **f**, **g** Tomograms of *caspQ* foci after chemically fixation (**f**) and high-pressure freeze-substitution (**g**), representative from tomograms of three individuals. Chemical fixation induces plasmolysis, allowing visualization of PM detachment and extracellular vesicles, while in high-pressure freezing apoplastic space is compressed and vesicles are still present but squeezed. Inside the *caspQ* structure, thin transversal aligned lamellae are detected by both methods. Scale bar 0.1 µm.

Next, we inspected the ultra-structure of *caspQ* dots by transmission electron microscopy (TEM) coupled with KMnO_4_ as a lignin stain. Here, we found that *caspQ* mutants display dramatic phenotypes in cell wall structure (Fig. 2a). At 1.8 mm from the root tip, wild-type CS appears as a thin lignified wall, sandwiched between the two tightly attached plasma membranes of neighboring cells (Fig. 2a, wild type). *caspQ* mutants, by contrast, initially displays a spectrum of defects, from strong plasma membrane detachment/non-adhesive with extracellular vesicles and irregular lignification (Fig. 2a, *caspQ* root 1) to irregular, lignified cell wall thickenings and less pronounced, but still observable membrane detachment (Fig. 2a, *caspQ* root 2). We found similar, but less frequent, membrane detachment and extracellular vesicles already in the triple *casp1 casp3 casp5*, but not in double *casp3 casp5* (Fig. 2b, c, Supplementary Fig. 4a, b). Moreover, *esb1* also displayed an intermediate phenotype with significantly thickened, lignified cell wall and enhanced membrane detachment (Fig. 2b, c, Supplementary Fig. 4a, b). By observing sequential developmental sections of the same seedling in wild-type and *caspQ* (Fig. 2d, e), we found that the variability of the *caspQ* phenotype was due to a developmental progression. In wild-type, the CS forms approximately at 1 mm from the root tip in 5-day-old seedlings. By capturing first images at 0.9 mm from the root tip in wild-type, we observed either of two stages: no difference in membrane or cell wall (stage 0), or invaginations and presence of extracellular vesiculo-tubular bodies (EVBs), but with no obvious cell wall thickenings yet (stage 1) at the predicted position of the CS. In follow-up sections (1.1 mm), some cells displayed a thin CS, flanked by invaginations and EVBs on the edges (stage 2). Finally, at 1.2 mm, we detected a wider, mature CS, with little to no EVBs on the flanks (stage 3) (Fig. 2d, e). To our knowledge, such an ultra-structural analysis of progressive CS development has not yet been reported, especially the association of EVBs with early stages of CS formation. In *caspQ*, we found early stage (stages 0 and 1) to resemble wild-type. In the following sections, however, a modified wall became apparent that displayed thickening in all directions (as opposed to the strictly confined, thin strip of the wild-type). Associated with this was a continued presence of EVBs and strong membrane detachment. We categorized this as mutant Stage 2 (stage 2 ^m^). Only in later sections, around 2.4 mm, EVB presence and membrane detachment finally abated with a strongly thickened, variably shaped cell wall at the position of the CS (Fig. 2b, d). We categorized this as mutant stage 3 (stage 3 ^m^), as the final stage of central cell wall formation in *caspQ* (Fig. 2d, e). In summary, this analysis revealed that *caspQ* appears to have a prolonged secretion phase, an inability of the plasma membrane to attach to the lignified wall and an uncontrolled cell wall outgrowth at the presumptive CS (Fig. 2c, d).

Chemical fixation for TEM always results in some degree of plasmolysis, which conveniently visualizes membrane attachment below the CS - and here, highlights the absence of attachment in *caspQ* (Fig. 2f, Supplementary movie 1). In order to observe *caspQ* membrane and cell wall in a turgid cell, without fixation artifacts, we generated TEM tomograms after cryo-fixation (Fig. 2g, Supplementary Fig. 4c). Here, the CSD membrane appeared turgid and not invaginated, but still separated from the wall proper by a compacted matrix containing extracellular membranes, appearing as bigger tubules or vesicles (Supplementary movie 2). Therefore, both EM methods detect the presence of EVBs and membrane separation in *caspQ*.

We additionally noticed that, especially in tomograms, *caspQ* cell wall domains appeared heterogenous after lignin staining with lighter, lamellae-like cell wall, alternating with darker, strongly stained regions (Fig. 2f, Supplementary movie 1). To understand the possible basis of this heterogeneity, we first blocked monolignol production by treating seedlings for 24 h with piperonylic acid (PA). This efficiently inhibits lignin deposition in de novo grown root sections, as evidenced by disappearance of KMNO_4_ staining (Supplementary Fig. 2). Yet, *caspQ* cell wall thickenings remained, indicating that they consist of a non-lignin cell wall matrix, which in turn becomes impregnated with lignin. We then wanted to see whether the lamellar structures in the mutant are due to an abnormal, “mixed” deposition of suberin and lignin. We therefore generated a pELTPxve»GELP12 line in the *caspQ* mutant background. GELP12 expression has previously been shown to abrogate suberin accumulation in the endodermis^25^ and we could observe the same effect in *caspQ* (Supplementary Fig. 5b). Nevertheless, lamellar structures in *caspQ* were found to persist in these lines, suggesting that they are not made of suberin (Supplementary Fig. 5c). We conclude that cell wall and membrane ultrastructure are dramatically affected in *caspQ*, associated with highly active and persistent secretion, as well as an extensive and disorganized cell wall deposition of lignin and additional, currently unknown matrix. Importantly, early CS development in wild-type shows a similar appearance of EVBs, but only in a restricted and transient fashion.

### caspQ foci overgrowth depends on SCHENGEN pathway activation

**Fig. 3:**
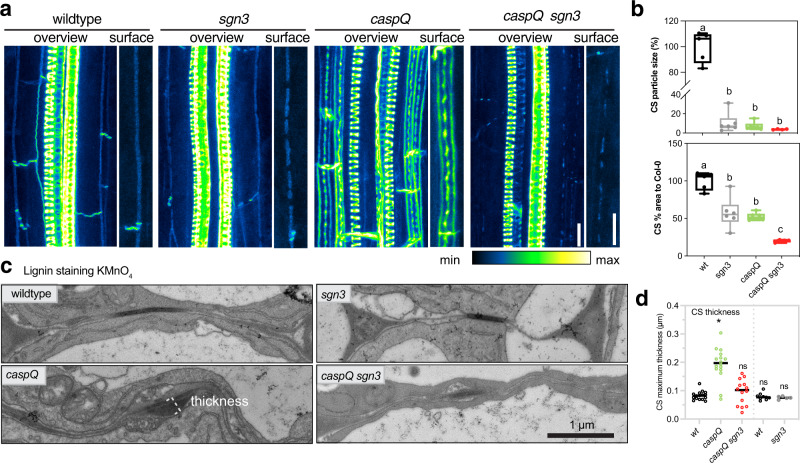
Outgrowth of caspQ lignin microdomains is SGN3-dependent. **a** Maximum intensity projections of lignin stained 5-day old roots at endodermal cell number 24 in wild-type, *sgn3*, *caspQ* and *caspQ sgn3*. Scale bars 10 µm. **b** Particle analysis of CS surface as described in Online Methods, with variables Average particle size (%) and CS % area to wild-type measured per 46 µm of CS surface. Boxplots with minima, median, maxima and data points (*n* = 6) and significant differences to wild-type shown with different letter by one-way ANOVA, Dunnett’s test *p* value < 0.05. **c**, **d** Electron micrographs of KMnO_4_ lignin-stained CS of 5-day old roots at 1.5 mm from root tip in wild-type, *sgn3*, *caspQ* and *caspQ sgn3* and respective quantification of CS maximum thickness stained by KMnO_4_ (**d**). Shown is the mean and data points from two experimental sets (separated by dashed line, first set *n* = 16 cell CS measurements from two individuals per genotype; second set *n* = 8 cells CS measurements from one individual per genotype). Statistical differences to wild-type one-way ANOVA, Dunnett’s test *p* value < 0.05.

The SGN3 receptor pathway monitors CS integrity, by detecting diffusion of stele-derived ligands into the cortical space. Complete absence of SGN3 signalling in SCHENGEN pathway mutants leads to incomplete fusion of the CS, whereas prolonged SCHENGEN pathway stimulation in other CS mutants, causes compensatory cell corner lignification and early suberization^13^. To test whether the observed *caspQ* phenotypes are due to SGN3-pathway activation, we targeted the *SGN3* locus by CRISPR-Cas9 in the *caspQ* mutant. As expected, mutating *sgn3* removed cell corner lignification (Fig. 3a) and early suberization (Supplementary Fig. 6) of *caspQ*. Yet, *caspQ sgn3* also showed severely reduced number and overgrowth of the central lignin foci (Fig. 3a, b). They appeared less numerous, smaller and more weakly stained in *caspQ sgn3* than in *caspQ* (Fig. 3a, b, Supplementary Fig. 7a). EM ultra-structure confirmed fluorescent lignin staining, since we observed *caspQ sgn3* lignin foci less frequently and, when present, they appeared thinner and lacked much of the enhanced wall deposition seen in *caspQ* mutants (Fig. 3c, d; Supplementary Fig. 6b). Moreover, *caspQ sgn3* revealed an additive phenotype of smaller and less abundant central lignin foci than either *caspQ* or *sgn3* single mutants. We interpret these foci as CS-initiation sites that are positioned and formed independently of CASPs and SGN3. CASPs and SGN3 then act together to promote the growth and fusion of these sites into a final fused strip.

### The lignin machinery still accumulates in central foci in caspQ

**Fig. 4:**
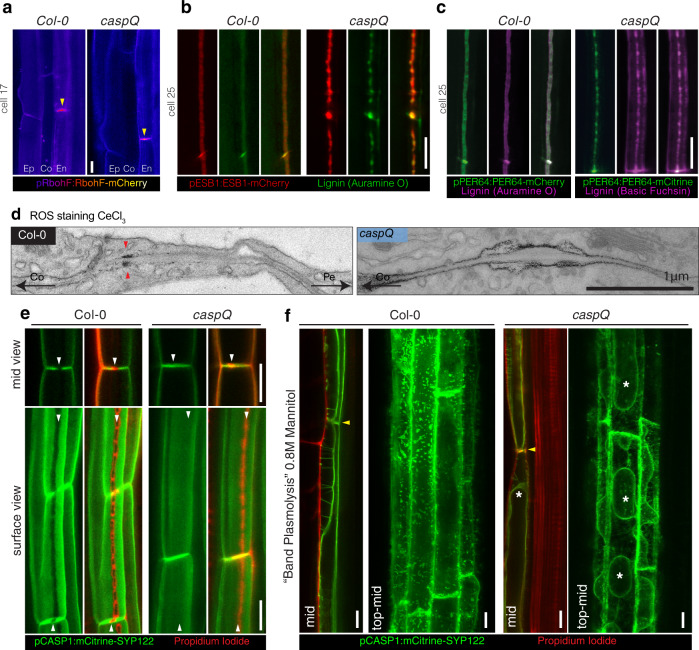
CASPs are not required for localized lignification but for CSD protein exclusion and cell-wall adhesion. **a–c** Localization of CS-lignifying enzymes in endodermis of 5-day-old seedlings of wild-type and *caspQ*: RBOHF-mCherry at cell 17 accumulation in CS (yellow arrow) Ep, epidermis; Co, cortex; En, endodermis (**a**); ESB1-mCherry and Auramine O-stained CS-lignin (**b**) and PRX64-Cherry with Auramine O stained CS-lignin (wild-type) and PRX64-mCitrine with Basic Fuchsin stained CS-lignin (*caspQ*) (**c**). Shown are pictures representative from three individuals and three independent experiments. **d** Electron micrographs from CeCl_3_ ROS-stained roots of wild-type and *caspQ*. Note the cortex side-stained CS in wild-type (red arrow), in contrast to the broader and plasma-membrane associated staining in *caspQ*. Shown is a representative picture from 16 cells from two individuals. **e** Plasma-membrane marker pCASP1::mCitrine-SYP122 is excluded from CSD labelled by the Propidium Iodide (PI)-stained CS in wild-type (see white arrows). In contrast, in *caspQ* pCASP1::mCitrine-SYP122 localizes to the CSD labelled by PI (white arrows). **f** Plasma membrane marker pCASP1::mCitrine-SYP122 and PI-stained cell-walls after plasmolysis in seedlings mounted in 0.8 M Mannitol solution. For wild-type in endodermal mid-view, plasma membranes of neighbor cells remain attached to the CS position (yellow arrow), forming the so-called band-plasmolysis; while in a maximum projection of top to mid Z-stacks membrane appears virtually all attached to the cell wall. For *caspQ*, in both mid and top-mid views the plasma membrane is detached from the CS cell wall (yellow arrow) and protoplasts can be distinguished (asterisks). Pictures in (**e** and **f**) are representative of three reproducible experiments with three individuals per genotype and treatment. Ep epidermis, Co cortex, En Endodermis, Pe pericycle. Scale bars 10 µm (except in **d**. scale bar 1 µm).

We have previously proposed a model whereby CASPs act as scaffold proteins, bringing together a membrane-localized NADPH oxidase (RBOHF), peroxidases, such as PER64 and other lignin-polymerizing proteins for efficient and localized lignin formation^7^. The full knock-out of CASP activities in the *caspQ* mutant now allowed us to directly test whether CASPs are required for localization of the known CS localized lignin-polymerizing components. We therefore introduced marker lines for the *RESPIRATORY OXIDATIVE BURST F* (*pRBOHF:RBOHF-mCherry*), *ENHANCED SUBERIN 1* (*pESB1:ESB1-mCherry*) and *PEROXIDASE 64* (*pPER64:PER64-mCitrine*) (Fig. 4a–c). To our surprise, all markers accumulated in centrally aligned microdomains in *caspQ*. RBOHF:mCherry accumulated in a central position (Fig. 4a). Likewise, ESB1 and PER64 secretion to the central domain was not affected in *caspQ*, but in the surface view both enzymes accumulated in dotty-pattern perfectly co-localizing to the central lignin foci of *caspQ* (Fig. 4b, c). Next, we looked at NADPH oxidase-mediated ROS production using CeCl_3_ staining and EM. Upon ROS presence, CeCl_3_ produces a precipitate detectable in EM^7,26^. In wild-type, precipitates are exclusively detected on the cortex side of the CS, presumably because CeCl_3_ cannot penetrate the CS. SGN3-pathway stimulation leads to enhanced and de-localized ROS production^13^. CeCl_3_ precipitates in *caspQ* are seen throughout the whole central plasma membrane, surrounding the central foci and spanning toward cortex and pericycle sides (Fig. 4d). Thus, CASPs are not required for localizing NADPH oxidase to the initial central foci, they appear to be needed to restrict and confine ROS production within the foci. Nevertheless, increased penetration of CeCl_3_ might at least partially account for the enhanced CeCl_3_ precipitates observed.

### CASPs are required for forming a protein exclusion zone and plasma membrane-wall attachment

In addition to the cell wall modification itself, the CS is characterized by the formation of a membrane domain, the CSD, that excludes most plasma membrane proteins and is tightly attached to the adjacent cell wall^6,10,22^. While CASP microdomain formation perfectly co-incides with the appearance of protein exclusion and membrane attachment, it could not be established whether CASPs are indeed required for these features. To test this, we introduced the plasma membrane marker *pCASP1::mCitrine-SYP122* into *caspQ* and found that the protein exclusion zone was completely absent. In wild-type, CSD exclusion visualized by mCitrine-SYP122 can be easily monitored by complementary localization with PI that stains CS in its early stages. In wild-type, mCitrine-SYP122 was strictly complementary to PI-stained CS (Fig. 4e). By contrast, mCitrine-SYP122 clearly overlapped with the strongly-PI stained, central lignin foci of *caspQ* (Fig. 4e). Next, we tested whether CSD attachment to the cell wall was affected in *caspQ*. When mounted in a hyperosmotic solution, the plasma membrane of wild-type seedlings retracts from periclinal walls but stays attached to the CS ring in the anticlinal walls, generating what is called a “band plasmolysis”^27^ across different cells in a mid-view, but no obvious plasmolysis in a top-to-mid view due to the lateral anchoring to the CS (Fig. 4f). In contrast, in *caspQ* we observed a plasmolysis typical for other cell types, with membrane detachment and protoplasts appearing in all views similar to one displayed in *myb36*, which lacks the entire CS differentiation program^28^ (Fig. 4f; Supplementary Fig. 8). These findings are consistent with our EM observations, where *caspQ* plasma membrane at lignin foci displayed detachment or invagination in early stages and resembled normal plasma membrane (with weak, fixation-induced detachments) at later stages (Fig. 2a–d). Altogether, these results show that CASPs are required to form the two defining features of the CSD, i.e., protein exclusion and membrane-cell wall attachment.

### A minimum of three CASPs are required of complementation of the quintuple mutant

**Fig. 5:**
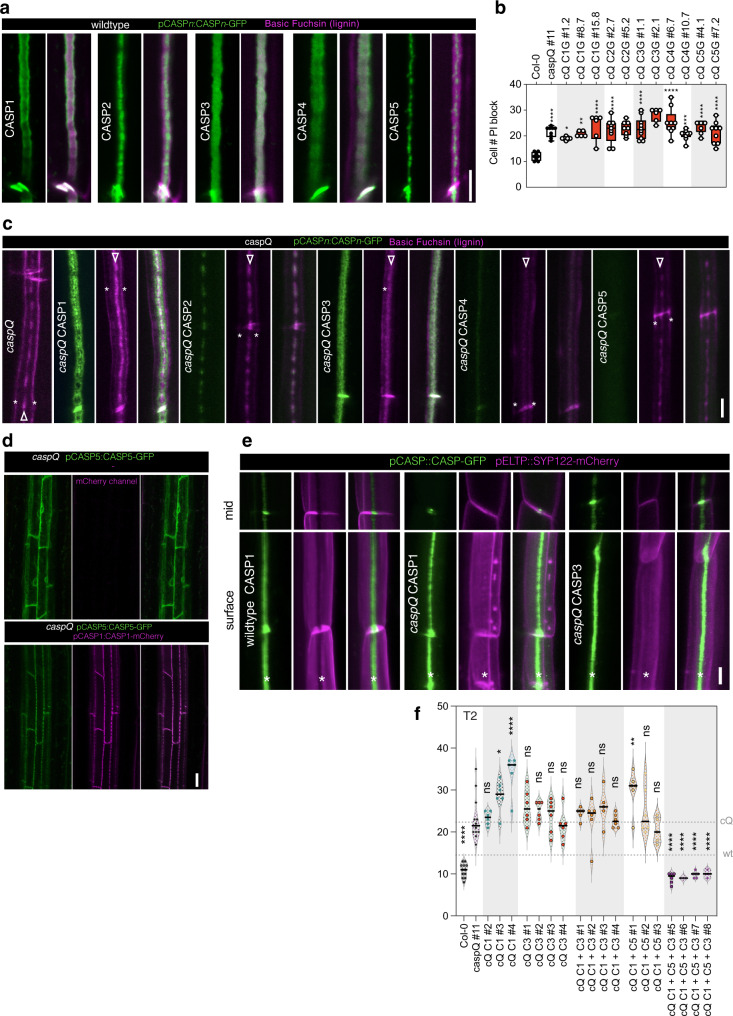
CASP1-CASP5 diverged in nanodomain localization and function to cooperate in Casparian Strip growth. **a** CASP1- to CASP5-GFP (green) nanodomain localization in CS surfaces stained with Basic Fuchsin (magenta) at endodermal cell number 15 of wild-type seedlings (pCASPn:CASP-GFP lines). Pictures are representative from three experiments. **b** Propidium iodide uptake assay in wild-type, *caspQ* and single *CASP1-5* complementation *caspQ* + pCASPn:CASP-GFP, shown as box-plots with 25–75th percentiles, mean, and whiskers of minima to maxima from *n* = 8 individuals, from at least two independent T3 homozygous lines per gene. Statistical differences by one-way ANOVA, Tukey’s test comparisons to wild-type (adjusted *p* < 0.05). **c** Detailed surface view of lignin staining with Basic Fuchsin and CASP1-5:GFP localization in *caspQ* and *caspQ* single *CASP1-5* complementation lines at endodermal cell number 25. Lignin stain at cell corners typical of CS-defective mutants (asterisks), and at presumptive CS (arrowheads). **d** pCASP5:CASP5-GFP in *caspQ* is retained in ER, but is able to reach CSD when co-expressed with pCASP1:CASP1-GFP in *caspQ*. **e** CSD exclusion zone (asterisk) in wild-type and *CASP1-* and CASP3- complementation *caspQ* + pCASPn:CASP-GFP visualized with plasma-membrane marker pCASP1:SYP122-mCherry. Neither CASP1-GFP, nor CASP3-GFP presence is sufficient to create a CSD exclusion for SYP122-mCherry in *caspQ* background. **f** Single, double and triple CASP1-5 complementation of *caspQ* obtained by combinatorial gateway constructs with one, two or three CASPs in T2 for a selection of constructs in individuals selected for presence of transgene, *n* = 6 individuals. Statistical differences by one-way ANOVA, Dunnet’s test comparisons to *caspQ* (*p* < 0.05). Pictures in (**c**–**e**) are representative from at least three independent experiments. Scale bars 5 µm (**a**, **c**, **e**) and 20 µm (**d**).

Having obtained a “blank slate” in the endodermis with respect to CASP activity also finally allowed us to test whether individual CASPs have distinct or redundant function. For this, we decided to reconstitute CASP function in *caspQ* by re-introducing CASPs one by one. When observing GFP fusions of CASP1-5 in wild-type at high resolution, each CASP displayed slightly different localization with respect to the CS. (Fig. 5a). CASP1 shows preferential localization at the edges of the strip^10^, while CASP2 and CASP5 appear in more central, dotted domains and CASP3 and CASP4 are localized in a homogenous band (Fig. 5a). These localization patterns reinforced the idea that CASP function is only partially interchangeable. Indeed, none of the single CASPs was able to restore the mutant phenotype when introduced into *caspQ* (Fig. 5b, c). Ectopic cell corner lignification in all lines indicated a defective CS, although CASP1-, CASP3- and CASP4-GFP *caspQ* complementing lines showed a more fused lignin distribution, with less individual foci. CASP2 and CASP5, by contrast failed to improve the *caspQ* lignin foci (Fig. 5c, Supplementary Figure 9a). All CASPs appeared to be less stable in caspQ than in wild-type, possibly due to their inability to form polymeric scaffolds. Interestingly, single CASP5-GFP appeared stuck in the ER and never reached the central plasma membrane domain in *caspQ* mutants (Fig. 5d). However, a CASP5-mCherry could be “mobilized” by co-expression with CASP1-GFP, in which case it reached the central plasma membrane domain, where it co-localized with CASP1-GFP (Fig. 5d). By combining CASP1- or CASP3-GFP in *caspQ* with a *caspQ* line containing a red plasma membrane marker, we found that neither CASP was able to form a protein exclusion zone, despite CASP1 and CASP3 appearing to form a band at the central plasma membrane domain (Fig. 5e). This suggests that interaction between several CASPs is needed to generate a CSD exclusion zone. By crossing *caspQ* CASP1-mCherry with *caspQ* CASP2 to 5-GFP we could demonstrate that also CASP double combination could not effectively rescue the *caspQ* PI phenotype (Supplementary Fig 9b). In order to identify a minimal CASP combination for rescue of *caspQ*, we generated a combinatorial Gateway collection of CASP genomic entry clones with endogenous promoters and terminators for triple Gateway. After generating single, double and triple combinations, we screened for PI block of individual T1 seedlings of each construct. We confirmed that single and double combinations cannot rescue *caspQ* (Supplementary Fig. 9c). Yet, one triple combination (CASP1, CASP3 and CASP5) efficiently complemented *caspQ* in most independent T1 individuals (Supplementary Fig. 9c). T1 complementation analysis was confirmed in a selected number of T2 lines (Fig. 5f). In summary, the five endodermal expressed CASPs of *Arabidopsis* present an evident degree of diversification, both in their nanodomain localization within the CS, as in their complementing activity. Our multiple complementation efforts suggest that a minimum of three CASPs is necessary to restore endodermal *caspQ* function, with a combination of CASP1, CASP3 and CASP5 being the most efficient in our hands.

### CASPs evict EXO70A1 from CS initiation sites

**Fig. 6:**
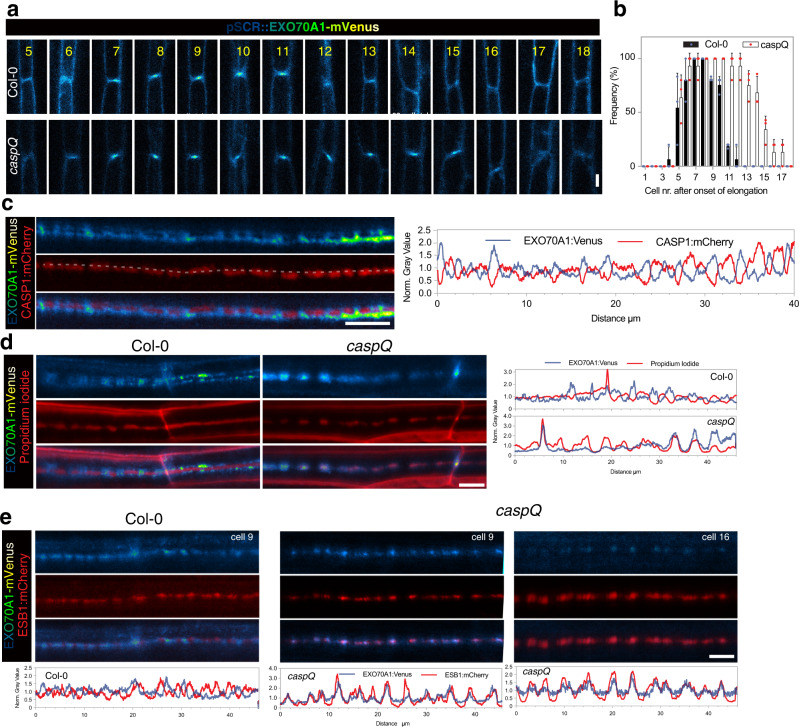
CASPs are necessary for EXO70A1 removal and progression in growing Casparian Strip. **a**, **b** Dynamic accumulation of EXO70A1-mVenus at CSD, inspected along consecutive endodermal cells (5–18) in wild-type and *caspQ*, confocal picture from representative individuals (**a**) Shown are the frequencies from three independent experiments (mean and standard deviation), each of which performed in *n* = 6 individuals (**b**). **c** Mutual exclusion of EXO70A1-mVenus and CASP1:mCherry in CS surfaces from wild-type seedlings at cell number 8. Shown is the profile for each channel obtained from same picture, drawing a line of 20 pt thickness (exemplified by dashed gray line). **d** Mutual exclusion (wild-type) and co-localization (*caspQ*) of EXO70A1-mVenus and Propidium iodide in CS surfaces in cells number 8–9 with respective profiles, obtained as in (**c**). **e** Mutual exclusion (wild-type, cells number 8–9) and co-localization (*caspQ*, cells number 9 and 16) of EXO70A1-mVenus and ESB1-mCherry in CS surfaces with respective profiles, obtained as in (**c**). Experiments (**c**–**e**) were repeated at least three times with same results. Scale bars 5 µm.

Immediately preceding the appearance of CASPs, EXO70A1 targets the exocyst complex transiently to the position of the incipient CSD, its knock-out leading to complete delocalization of CASP secretion^16^. Since *caspQ* appears to lack a CSD, but is nonetheless able to form correctly localized, albeit aberrantly structured lignin foci, we investigated how the exocyst complex localized in the mutant. We monitored the appearance of EXO70A1, the subunit responsible for exocyst localization and assembly^16,29^, in wild-type and *caspQ* using an EXO70A1 line with endodermis-specific expression (*pSCR::EXO70A1-mVenus*). Endodermis-specific expression of EXO70A1 was used to improve imaging and allow cell surface observations. As reported for the native promoter line, *pSCR*-driven EXO70A1 accumulated transiently at CSD in wild-type^16^ (Fig. 6a, b). Despite being weakly expressed from the early-endodermis *SCR* promoter, we were able to precisely image EXO70A1 dynamic at the endodermal surface. Interestingly, when combined with *pCASP1::CASP1-mCherry*, a mutually exclusive localization of EXO70A1-mVenus and CASP1:mCherry was observed in wild-type (Fig. 6c). EXO70A1 accumulated in the gaps between CASP1-mCherry at the string-of-pearls stage and later surrounded the strip, as if EXO70A1 was excluded from where CASP domains have formed, accumulating instead in spots where CASP1 is still to be delivered, i.e., the gaps of the string-of-pearls stage and the edges during CS widening. This dynamic was consistent with the mid-section views, where EXO70A1 precedes CASP1 appearance, but then disappears from CASP1-mCherry containing spots, such that the two proteins hardly co-localize^16^. Thus, CASP and EXO70A1 localization appear to be mutually exclusive. Intriguingly, in *caspQ*, the same initial localization of EXO70A1 was observed. Yet, EXO70A1 then showed a systematically prolonged persistence at the central foci in the mutant (Fig. 6a, b). Moreover, when co-stained with PI in order to highlight lignin, we found that EXO70A1 localization strongly co-incided with the lignin foci (Fig. 6d). Intriguingly, in wild-type, EXO70A1 was again excluded from the PI-stained domains at the string-of-pearls stage, showing an opposite pattern to the *caspQ* mutant. We next introduced pESB1:ESB1-mCherry to monitor a bona fide CS marker that could be used in both backgrounds. Here again, we observed a negative correlation of ESB1 and EXO70A1 localization in wild-type and a strictly positive correlation of the two proteins in *caspQ*.

In summary, the mutually exclusive localization of CASP and EXO70A1, together with the fact that EXO70A1 persists at the lignin foci of *caspQ*, indicates that CASPs act to evict EXO70A1 from the forming CS domains, thereby forcing its re-localization to the edges and into the remaining gaps.

### CASP1-proximity labelling identifies novel, CS-associated Rab GTPases

**Fig. 7:**
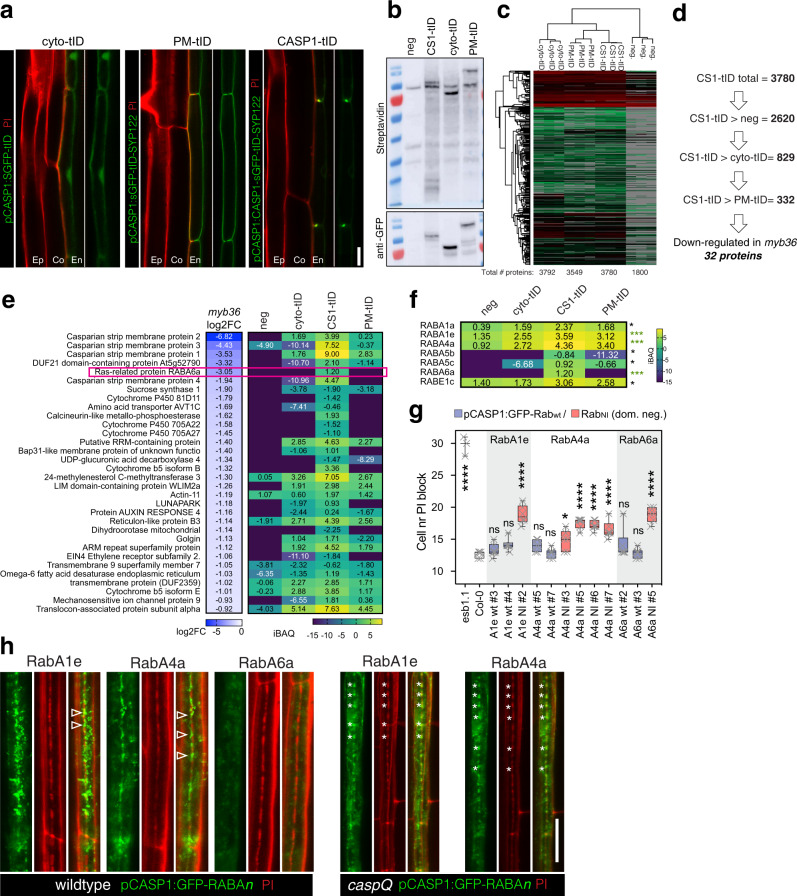
CASP1-proximity labelling identifies RabAs with CASP-dependent CS-localization and function in CS integrity. **a** Endodermal expression and sub-cellular localization of GFP-turboID protein fusions: cytosolic (*cyto-tID*, pCASP1:sGFP-turboID), plasma membrane (*PM-tID*, pCASP1:sGFP-turboID-SYP122) and CS-localized (*CASP1-tID*, pCASP1:CASP1-sGFP-turboID). Pictures are representative from at least three independent experiments. **b** Western Blots of roots of *cyto-tID*, *PM-tID* and *CASP1-tID*, negative control (pCASP1:CASP1-GFP) grown for 6d in normal media and additional 1 day in biotin (50 µM) containing plates; showing biotinylation pattern and presence of intact turboID fusion constructs. Similar biotinylating patterns were observed in at least three independent pilot experiments, shown are the pooled replicates input samples used for LC/MS before purification. Molecular weight of red marker is 70 kDa for both blots. **c** Cluster Analysis of LC/MS-MS identified proteins from streptadividin purified root extracts as in (**b**) done in Perseus, from three biological replicates of *cyto-tID*, *PM-tID*, *CASP1-tID* and negative control. **d** Sequential pairwise analysis (*t*-Test, two-sided, Bonferroni adjusted *p* value < 0.05 and log2iBAQ differences above 0 for maximum inclusiveness) reveal 332 significantly enriched proteins in *CASP1-tID* relative to all other samples. From those 332 proteins, 32 are significantly down-regulated in mutant of CS-transcriptional regulator *myb36* (based on Liberman et al., 2015). **e** Transcriptional downregulation in *myb36* (log2-fold change) and protein abundance (iBAQ) in turboID experiment of the 32 proteins significantly enriched in CS1-tID samples and significantly downregulated in *myb36*, highlighting Ras-related small GTP-ase, RabA6a. **f** Significantly-enriched (as in **d**) Ras-related small GTPases in *CS1-tID* sample, ***p < 0.01 and *p < 0.05. **g** PI uptake assay of endodermal specific overexpression of the three most enriched RabAs (A1e, A4a and A6a, enriched in CS1-tID *p* < 0.01) as wild-type sequence and as dominant negative N-I mutants, wild-type and *esb1-1* as reference, shown as box-plots with 25–75^th^ percentiles, mean, and whiskers of minima to maxima from *n* = 6 individuals per genotype. Statistical differences by one-way ANOVA, Tukey’s test comparisons to wild-type (adjusted *p* < 0.05). **h** Localization of the three GFP-RabAs in wild-type and *caspQ* (with exception of RabA6a, where no lines with detectable signal could be retrieved) in cell number 9 stained with PI. Note the presence of GFP-Rab signal intercalated with PI-stained CS string-of-pearls (arrowhead) in wiltype, in contrast with perfect co-localization of GFP-Rab with PI stained central foci of *caspQ* (asterisks). Experiment was repeated at least three times with same results. Scale bars 10 µm.

CASPs are very small transmembrane domain proteins with limited surfaces for biochemical functions. In our efforts to understand CASP function, we envisioned that identifying CASP-interactors could help us understand how these small proteins play such a critical role in CS development. Previous attempts of identifying CASP1-GFP interactors by co-immunoprecipitation only identified CASP3^6^. This was attributed to low CASP1-GFP solubility, due to strong attachment of the CSD to the cell wall. We therefore decided to employ proximity labelling, fusing CASP1 to the TurboID biotin ligase (*pCASP1::CASP1-sGFP-TurboID*, short CASP1-tID). TurboID generates highly reactive biotin-AMP that covalently attaches to proximal proteins within a defined radius. This allows a broader detection of both strong and weak interactors, since biotinylation occurs in vivo, allowing stringent washing and increased solubilization of tightly CS-associated proteins upon extraction. Since the CS is not a membrane-confined compartment, we generated two TurboID controls expressed under the same endodermal promoter: a cytosolic and a plasma membrane TurboID (*pCASP1::sGFP-TurboID*, cyto-tID and *pCASP1::sGFP-TurboID-SYP122*, PM-tID), allowing us to filter for abundant proteins that would become labelled due to random contacts. Thanks to the GFP tag we could ascertain correct expression, localization and integrity of the fusion proteins (Fig. 7a, b). After biotin feeding, three lines showed a pool of diverse biotinylated proteins (Fig. 7b). Three biological replicates were purified and analyzed by LC-MS/MS-Spec, with pCASP1::CASP1-GFP plants as negative control^30,31^. In clustering and PCA analyses, the biological replicates cluster together, with CASP1-tID being closer to the PM-tID than the cytosolic control (Fig. 7c). As expected, self-biotinylated GFP-tID and respective fusion proteins scored among the most abundant proteins in TurboID samples. Moreover, GFP-tID abundance was similar in intensity among replicates and tID samples, implying labelling and purification yields were comparable, and allowing to confidently compare iBAQ values between the distinct transgenic lines (for iBAQ values see Materials and Methods).

We then checked if known players involved in CS formation were detected and how they behave in different turboID samples (Supplementary Fig. 10). Besides CASP1, we detected CASP2, CASP3 and CASP4, specifically enriched in CASP1-tID. CASP5, the smallest CASP protein, although exclusively detected in CASP1-tID, did not pass the quality criteria for LC-MS/MS-identification, i.e., to be identified by more than one peptide, thus could not be included in the analysis. Members of the EXOCYST complex were detected in turboID samples, but with no preference for CASP1-tID sample, consistent with their transient accumulation to the CSD and otherwise broad localization to the plasma membrane, and the complex’s broad function in membrane trafficking^16,29,32^. Notably, we identified one or two members of each subunit of the complex (SEC3,5,6,8 and 15,84,70), with exception of SEC10 for which no isoform was identified.

From the SCHENGEN signalling pathway, we identified SGN1, RBOHF and RBOHD as specific to CASP1-tID and PM-tID samples. This is expected, as none to these components are CSD-specific and only RBOHF shows some enrichment to this domain (Fig. 4a)^7,13^. Moreover, the PM-tID protein occupies a manyfold bigger surface, compared to CSD localized CASP1-tID^7,13^, which should only allow CSD-specific, or highly enriched proteins to be preferentially detected in the CASP1-tID sample. Expectedly, we did not detect CS-localized lignification enzymes, e.g., ESB1, PER64, UCC^7,9,10^, since their apoplastic localization separates them from the biotinylation reaction occurring on the cytosolic side of the plasma membrane.

Next, we filtered for CASP1-tID enriched proteins (Fig. 7d). Filtering the initial 3780 proteins against negative control, cytosolic and plasma membrane samples generated a set of 332 proteins significantly enriched in the CASP1-tID sample (Supplementary Data 2). After this filtering, CASP1 appears as the most abundant protein, followed by ubiquitin; CASP2,3 and 4 appear in the top 50. A survey of the list reveals proteins associated with protein degradation. Besides Ubiquitin itself, 26 S proteasome subunits, members of ESCRT complex, TOM-like proteins, and FREE1/FYVE^33^. Moreover, three Arabidopsis sterol methyl transferases rank among the top ten and proteins related to monolignol biosynthesis and oxidation, e.g., cytochrome P450, were also found in the list (Fig. 7e, Supplementary Fig. 10)^34^. Sub-cellular localization analysis revealed a striking prevalence of ER-localized proteins in the CASP1-tID enriched fraction (Supplementary Fig. 11).

Intriguingly, when filtering for vesicle transport regulators, we observed a clear overrepresentation of a subclass of Rab proteins, called the RabA class. Of the 57 *Arabidopsis* Rab-GTPases^35^, we detected 36 members in the whole experiment, with seven being specific to the CASP1-tID sample (Supplementary Fig. 12). One of them, RabA6a, additionally showed decreased expression in *myb36* (Fig. 7e, f). Rabs are central regulators of vesicle fusion and membrane organisation and the RabA subclass (mammalian Rab11/25 subclass homologs) in particular is known to be involved in late steps of secretion or endosomal recycling^35^. Moreover, Rabs are known activators of the exocyst complex^36^. We therefore tested whether Rabs play a role in CS formation by generating cell-type specific overexpressors of dominant-negative Rab variants. Indeed, dominant-negative lines of the three most significantly enriched (*p* < 0.01) RabA homologs (A1e, A4a and A6a) showed a weak, but consistent delay in the formation of the apoplastic barrier, suggesting a role in CS formation and possibly CASP trafficking (Fig. 7g). Moreover, localization of two RabAs expressed under an endodermis-specific promoter, showed RabA-positive vesicular structures to be in close vicinity, but distinct from, initiating CS patches in wild-type. The *myb36*-regulated RabA6a signal was very weak, and we could not detect specific CS accumulation. By contrast, these RabA-positive compartments strongly overlap with cell wall foci in *caspQ* (Fig. 7h). Although localized to vesicular structures and not membrane domains, this situation closely resembles that of EXO70A1 described earlier and is consistent with RabAs acting as activators of exocyst during targeting of CASP-bearing vesicle to the central domain.

## Discussion

CASPs and CASP-LIKES represent a plant-specific branch of the MARVEL superfamily, a highly sequence divergent family of four trans-membrane spanning proteins^20,37^. Their presence not only in ophistokonts (animals and fungi), but also in plants, as well as heterokonta (oomycetes, brown algae) suggests that they are a truly ancient invention of eukaryotic cells^20^. Interestingly, their conservation is largely restricted to their overall topology (four transmembrane domains with termini inside) and few specific features of the transmembrane domains. MARVEL proteins display low conservation and independent evolution of complex sub-families in animals and plants. *A. thaliana*, for example, has 5 subfamilies with a total of 39 members, whereas *H. sapiens* has 4 subfamilies for a total of 27 members^20,38^. Nevertheless, there are tantalizing parallels in supposed functions of these proteins in different cell types. In animals, the four subfamilies are involved in seemingly divergent processes, such as regulation of tight-junction structure and functionality, regulation of pre-synaptic vesicle release, subdomain dynamics and signaling in immune cells, or cancer development^39–42^. In attempts to formulate an overarching, basic cellular function, it was pointed out that animal MARVEL proteins are often found to be highly abundant, conserved (i.e., potential orthologs are found in insects and nematodes) and associated with the formation of plasma membrane subdomains and/or late secretory events^40^. Surprisingly, in animals, even higher-order mutant and sometimes presumably full knock-outs of their respective functions, often leads to comparatively subtle phenotypes. Double mutant of occludin and tricellulin, for example, lead to changes of branching patterns of tight-junction strands, without interfering with their presence or overall functionality^40^. The same applies for presumably full knock-out of synaptic MARVELs, which does not fundamentally interfere with synaptic transmission, but merely alters probability of synaptic vesicle fusion under certain conditions^39^. A strong knock-down of MAL function, by contrast, leads to rather strong defects in the formation of a plasma membrane microdomain, the immunological synapse, as well as signalling in T-cells^42^. In budding yeast, a similar function in microdomain assembly and exclusion of endocytic markers was observed for the MARVEL protein Nce102^43,44^.

**Fig. 8:**
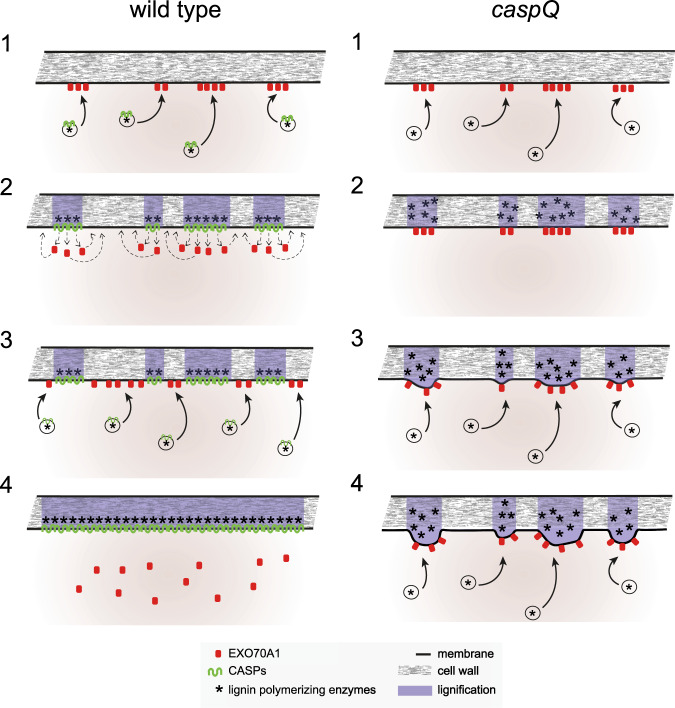
Conceptual model of CASP action in the endodermis. In presence of CASPs (*wild-type*): Vesicles carrying CASPs and lignin polymerizing enzymes are specifically targeted to EXO70A1 landmarks at the endodermal plasma membrane (1). Targeting specificity is possibly mediated by the presence of specific RabAs on the vesicles (not shown). CASPs form stable microdomains by polymerization, and mediate the nano-scale organization of lignin polymerizing enzymes in the cell wall. In addition, CASPs mediate, directly or indirectly, the eviction of EXO70A1 from the cytosolic membrane face. EXO70A1 re-locate to parts of the central region that are still free of CASPs (2). New CASP carrying vesicles deliver CASPs to the remaining gaps between microdomains marked by EXO70A1 (3), eventually fusing individual microdomains into a contiguous band and thus sealing the cell wall space (4). In absence of CASPs (*caspQ*): Vesicle carrying lignin polymerizing enzymes are correctly targeted to EXO70A1 landmarks (1), forming initially normal lignin foci in the correct position (2), absence of CASPs allows EXO70A1 landmarks to persist in the same initial position, further attracting vesicles and lignin polymerizing activity to the same foci, leading to their thickening (3). Continued, persistent secretion to the same foci eventually leads to strongly thickened, aberrantly structured lignin foci that are unable to fuse with each other and do not seal the cell wall space (4).

Our full knock-out of the endodermis-expressed CASPs, now provides one of the clearest phenotypes in multi-cellular eukaryotes and could serve as a paradigmatic example for MARVEL function beyond plants. Our data indicate that CASP-driven microdomain formation at the plasma membrane suppresses further secretion at a given site by evicting EXO70A1, the factor that is central for targeted secretion of CASP-bearing vesicles in the first place. The consequence of this negative feedback loop is to enforce continual displacement of EXO70A1 secretory foci along the median zone, leading to rapid filling of gaps in the initial string of microdomains and their eventual fusion into an uninterrupted band (Fig. 8). Inhibition of exocytosis, and possibly endocytosis, could be a critical feature to maintain CSD identity and membrane-wall attachment, i.e., the CSD could be a relatively inert surface for trafficking and protein diffusion.

The second aspect of CASP function lies in the feature of the microdomain itself. In the absence of CASPs, two hallmarks of the Casparian strip domain, membrane-attachment and membrane exclusion zone formation are clearly absent. Thus, in plants, MARVEL proteins are not only required for functions mediated by occludins and claudins in animals, but also by the adherens junction-forming cadherins. Finally, CASP domains are clearly necessary to form the homogenous, dense and spatially confined lignification that gave rise to the term “strip”, from the German “Streifen”, describing its flat appearance and well-defined width. In the quintuple CASP mutant, centrally focused delivery of the cell wall modifying enzymes is still occurring, but the resulting cell wall is clearly disorganized, of variable shape, excessive thickness and displaying currently unidentified, lamellae-like inhomogeneities within the lignified wall. Thus, although not required for enzyme localization at the cellular scale, CASPs do appear to be necessary to organize enzymatic activities at the nanoscale. The ability to generate stable protein platforms in the PM, that can then organize extracellular activities, engage in cell wall/matrix interactions or cell-cell contacts, is critical for cells in complex cellular communities. Possibly, this ability might be mediated by MARVEL proteins and might represent the elusive, encompassing role of MARVEL proteins in eukaryotes.

Finally, the strong and sustained secretion to a few median foci in the CASP quintuple mutants also revealed the presence of extracellular vesicles below the lignifying wall. Indeed, their presence in the mutants prompted us to look for EVBs during Casparian strip formation in wild-type, where we found EVBs to also be present, but only very transiently. The presence of EVBs is intriguing, considering that current models for the apoplastic delivery of lignifying components postulate passive diffusion or transporter-mediated transport of monolignols^45,46^ and lignifying enzymes to be transported by the canonical secretory pathway. Moreover, a recent report described very similar structures to be associated with suberisation in the endodermis^47^. Since we have no indication that either *caspQ* lignin foci or wild-type Casparian strips contain suberin, we conclude that EVBs in the endodermis are not associated only with suberisation, but also lignification and might be regarded generally as being associated with strong, focused secretion in non-elongating cells.

The plant exocyst complex clearly appears to have evolved a divergent and more complex role in plants than the one described in animals and fungi. The presence of 23 different EXO70 subunits suggested that the plant exocyst is not a general component required for tethering of any plasma membrane bound vesicles, but that numerous, distinct exocyst holocomplexes exist, whose role could be to mediate tethering of specific vesicle subpopulations, to different plasma membrane subdomains^17^. Yet, this model requires a matching complexity on the vesicle side that would allow to “read” different EXO70 landmarks, such that a given vesicle could be preferentially tethered to a specific EXO70 landmark. Intriguingly, plants present an additional significant radiation not observed in animals, the presence of 26 RabA GTPases, compared to only 3 Rab11/25 (RabA homologs) family members in mammals^35^. Here, we found that CASPs are in specific, close proximity to RabA family members, that RabAs localize to the central domain defined by EXO70A1, immediately suggesting that RabA-bearing vesicles might have specific affinity for EXO70A1 marked domains. Indeed, cell-type specific, dominant-interference lines of different RabA homologs collectively showed that RabAs have some function in CS formation. In animals and yeast clear evidence points to exocyst being a “Rab effector”, i.e., the exocyst being activated by Rabs (e.g., Sec4 in yeast, Rab11 in animals)^36,48^. It is therefore intriguing to speculate that plant cells could produce different classes of late secretory vesicles, discriminated by different RabA subclasses and that these vesicles have preferences for specific EXO70 subunits thus allowing for tethering of different vesicle populations to distinct plasma membrane subdomains. This would represent a very different solution to generate more complex targeting to plasma membrane subdomains than the one found in animals. In the case of CS formation, CASPs and other CS proteins might be segregated into specific RabA-bearing secretory vesicle populations, that would then preferentially tether to PM domains with an EXO70A1 mark.

Our analysis has revealed the as yet mysterious function of CASP proteins, i.e., formation of a protein exclusion zone, mediating membrane-wall adhesion and eviction of secretory tethering factors, all of which are required for proper CS membrane-wall sealing and microdomain fusion. The appearance of CASPs and occludins in analogous structures, i.e., CS and tight junctions, is an intriguing example of convergent evolutionary recruitment of a pre-existing functionality. Further mechanistic dissection of how CASPs execute these apparently transversal MARVEL functions, in such an amenable genetic and cellular system as the root endodermis, could serve as a paradigm for understanding MARVEL proteins from many different organisms and cell types. In plants, this study will enable further exploration of the plant-specific CASP-LIKE family of MARVELs, expressed in many cell types with diverse cell wall modifications and possibly unique membrane domains, e.g., suberization, abscission zone formation or pathogen-induced lignin deposition^24,49,50^.

## Methods

### Plant material and growth conditions

For all experiments, *Arabidopsis thaliana* (ecotype Columbia) was used. Seeds were surface-sterilized, sown on plates contained half-strength Murashige and Skoog (MS) + 0.8% Agar (Roth) medium, stratified at 4 °C and darkness for 3 days, and grown vertically in growth chambers at 21 °C at constant light for 5 days, unless otherwise stated.

All plant materials used, either published or here obtained are listed in Supplementary Data 1. The following available mutants were used *casp1-1* (SAIL_265_H05), *casp3-1* (SALK_011092), *casp5-1* (SALK_042116), *esb1-1*^9^, *sgn3-3* (SALK_043282) and *myb36-2* (GK-543B11). Novel mutant alleles created in this study using CRISPR-Cas9^21^ (see protospacer sequences in Supplementary Data 1) are *caspQ, caspQ sgn3, caspQ caspl-7ple*, using targeting constructs described below.

The following published constructs were introduced by floral dipping in *caspQ: pCASP1::CASP1-GFP, pCASP2::CASP2-GFP, pCASP3::CASP3-GFP, pCASP4::CASP4:GFP, pCASP5::CASP5-GFP*^6^*, pCASP1::mCit-SYP122*^14^*, pESB1::ESB1-mCherry*^9^*, pRBOHF:mCherry-RBOHF* and *pPER64::PER64-mCherry*^7^*; pCASP1::mVenus-SYP122* and *pELTP::3xmCherry-SYP122*^51^*; pCASP1::CASP1-mCherry*^16^ *and pELTPxve::GELP72*^25^.

### Cloning and plasmid construction

The In-Fusion Advantage PCR Cloning Kit (Clontech) and Gateway Cloning Technology (Invitrogen) were used for generating lines described thereafter. All constructs were transformed by heat shock into Agrobacterium tumefaciens GV3101 strain and then transformed into plants by floral dipping.

CRISPR-Cas9 targeting of genes *CASP1, CASP2, CASP4, SGN3, CASPL1-A1, B1, B2, C1, C2, D1* and *D2* were generated as reported in^21^. Briefly, forward and reverse primers containing protospacer sequences flanked by overhanging sites for each entry vector were annealed and ligated into entry clones. Alternating U6-26::gRNA and U3::gRNA containing vectors were assembled into intermediate vector at desired combinations by Golden-Gate reaction. Final vectors containing gRNA multiplexed ORFs, pUbi::Cas9 and FastRed selection were then generated by Gateway reaction. In Supplementary Data 1, protospacer sequences are listed in *Primer* list, final multiplexing vectors in *Plasmid* list and the resulting homozygous plant alleles listed and described in *Plants* list.

The pCASPL*n*::CASPL*n*-mCitrine constructs were generated by Infusion as follows: CASPL promoter and gene sequences were amplified with overlapping primers to mCitrine and introduced by Infusion into destination vector containing FastRed cassette (see plasmid and primer lists in Supplementary Data 1).

To generate single, double and triple CASP1-CASP5 combination constructs, CASP genes (promoter, gene and 3’UTR and terminator sequences) and GFP and/or mTurquoise tag were amplified by PCR with overlapping primers and cloned by Infusion into linearized gateway entry vectors, for any of 3 positions of Multi-site triple Gateway. Triple LR reactions were performed with different combinations of *CASP1* to *CASP5* genes, and in case of single and double CASP combinations dummy entry vectors were used at remaining positions. All construct combinations and respective primers are listed in Supplementary Data 1.

The pSCR::EXO70A1-mVenus construct was generated by LR from available entry vectors^16^.

For TurboID constructs, the sequences were amplified using primers listed in Supplementary Data 1, from vectors kindly provided by P. Moschou^52^, for BP reaction to Gateway entry clones, and translational fusions were performed with Multi-site Gateway reactions.

The pCASP1::GFP-RabA*n* constructs were generated by triple Gateway. Briefly, Rab-GTPases were amplified and introduced by BP reaction in pDNR p2r-p3. Dominant negative mutations (N-I) were introduced by PCR site-directed mutagenesis in respective entry vectors pDONR p2r-p3.

### RNA extraction and qRT-PCR

For RNA extraction, seedlings were grown for 5 days on half strength MS on mesh to facilitate root cutting. Approximately 60 mg roots were cut and frozen in liquid nitrogen and stored at −80 °C. Total RNA was extracted using a TRIzol-adapted ReliaPrep RNA Tissue Miniprep Kit (Promega). Reverse transcription was carried out with PrimeScript RT Master Mix (Takara). All steps were done as indicated in the manufacturer’s protocols. The qPCR was performed on an Applied Biosystems QuantStudio3 thermocycler using a MESA BLUE SYBR Green kit (Eurogentech). Transcripts are normalized to Clathrin adaptor complexes medium subunit family protein (AT4G24550) expression. All primer sets are indicated in Supplementary Data 1.

### Confocal laser-scanning microscopy

Confocal laser-scanning microscopy images were obtained using either a Zeiss LSM 880 (with Zen 2.1 SP3 Black edition), Leica SP8-X (with LasX 3.5.6.21594) or Leica Stelaris 5 (LAS X (2020) version 4.1.23273) microscopes. The following excitation and detection windows were used: GFP, Fluorol yellow: 488 nm, 500–530 nm; mVenus, mCITRINE: 514 nm, 505–530 nm; Propidium iodide: 561 nm, 590–650 nm; Calcofluor White: 405 nm, 430–485 nm; Basic Fuchsin: 561 nm, 600–630 nm.

Propidium iodide penetration assay was performed as described previously^22^. Briefly, seedlings were stained in PI (10 µg/mL) dissolved in water for 5 min and mount in same solution for imaging, with above defined settings. Scoring of endodermal cell number was initiated from onset of elongation, (defined as endodermal cell length being more than two times than width in the median, longitudinal section) until PI could not penetrate into the stele.

Lignin and cell wall staining was performed in fixed and cleared seedlings, following ClearSee-adapted protocol^13^. Briefly, 5-day-old seedlings were fixed in 3 ml 1 × PBS containing 4% paraformaldehyde for 1 h at room temperature in 12-well plates and washed twice with 3 ml 1 × PBS. Following fixation, the seedlings were cleared in 3 ml ClearSee solution under gentle shaking. After overnight clearing, the solution was exchanged to new ClearSee solution containing 0.2% Fuchsin and 0.1% Calcofluor White for lignin and cell wall staining, respectively, and incubated overnight. Next day samples were rinsed with fresh ClearSee solution for 30 min with gentle shaking, 2–3 times, and at least once overnight before observation.

Suberin was stained with Methanol-based Fluorol Yellow protocol^13^. Briefly, 5 day-old seedlings were fixed and cleared in methanol for at least 3 days at 4 °C, exchanging the methanol at least once. The cleared seedlings were transferred to a freshly prepared solution of Fluorol Yellow 088 (0.01%, in methanol) and incubated for 1 h. The stained seedlings were rinsed in fresh methanol and transferred to a freshly prepared solution of aniline blue (0.5%, in methanol) for counterstaining for 1 h. Finally, the seedlings were washed for 2–3 min in water before imaging. For *caspQ* pELTPxve»GELP72 assay, seeds were sown directly in half-strength MS containing 10 µM beta-estradiol, including control *caspQ*.

For plasmolysis, seedlings were firstly stained in PI (10 µg/mL) for 5 min, and directly mount in a 0.8 M mannitol solution just before imaging.

### Image analysis: CS particle analysis and surface profiles

All images were processed and analyzed in FIJI. All images were acquired with Leica SP8 or Stelaris at the described endodermal cell number with fixed dimensions: 63× objective, 4× optical zoom, 1024 × 512 pixel, such that CS length is approximately equal among samples, i.e., 46 µm. At desired cell number, a region with most flat CS surface was chosen, and a Z-stack was acquired with as many stacks required to capture whole strip in focus. For each image, stacks were projected with Maximum Intensity projection and processed as follows.

For CS particle analysis, images were then analyzed with the “Analyze Particles” FIJI plugin as follows: a segmented line of 50 pt was drawn along the CS and straightened to make a ROI. The Threshold “InterModes” was applied to straighten strip, and plugin “Analyze Particles” run. Shown is *Particle number* per ~46 µm of CS length; *Particle area* (µm^2^) and *CS area (%)* to wild-type at cell 20.

For CS surface profiles, a segmented line of 20 pt width was drawn along the strip, and a profile was obtained for each channel. In Excel, for each channel intensities were normalized to average intensity and plotted in GraphPad.

### Classical TEM analysis

Plants were fixed in glutaraldehyde solution (EMS, Hat- field, PA) 2.5% in phosphate buffer (PB 0.1 M [pH 7.4]) for 1 h at RT and postfixed in a fresh mixture of osmium tetroxide 1% (EMS, Hatfield, PA) with 1.5% of potassium ferrocyanide (Sigma, St. Louis, MO) in PB buffer for 1 h at RT. The samples were then washed twice in distilled water and dehydrated in ethanol solution (Sigma, St Louis, MO, US) at graded concentrations (30%–40 min; 50%–40 min; 70%–40 min; 100%–2 × 1 h). This was followed by infiltration in Spurr resin (EMS, Hatfield, PA, US) at graded concentrations (Spurr 33% in ethanol − 4 h; Spurr 66% in ethanol − 4 h; Spurr 100%–2 × 8 h) and finally polymerized for 48 h at 60 °C in an oven. Ultrathin sections of 50 nm thick were cut transversally at 1.8 mm from the root tip using a Leica Ultracut (Leica Mikrosysteme GmbH, Vienna, Austria), picked up on a copper slot grid 2 × 1 mm (EMS, Hatfield, PA, US) coated with a polystyrene film (Sigma, St Louis, MO, US). For sequential cut, the roots were cut every 0.1 mm starting from 0.9 mm from the root tip. Sections were post-stained with uranyl acetate (Sigma, St Louis, MO, US) 4% in H_2_O for 10 min, rinsed several times with H_2_O followed by Reynolds lead citrate in H_2_O (Sigma, St Louis, MO, US) for 10 min and rinsed several times with H_2_O. Micrographs were taken with a transmission electron microscope Philips CM100 (Thermo Fisher Scientific, Waltham, MA USA) at an acceleration voltage of 80 kV with a TVIPS TemCamF416 digital camera (TVIPS GmbH, Gauting, Germany) using the software EM-MENU 4.0 (TVIPS GmbH, Gauting, Germany). Panoramic alignments were performed with the software IMOD^53^.

### High pressure freezing and cryo-substitution

For the High Pressure Freezing, pieces of root 5 mm long were cut from tip, and then placed in an aluminum planchet of 6 mm in diameter with a cavity of 0.1 mm (Art.610, Wohlwend GmbH, Sennwald, Switzerland) filled with 15% Dextran in 2-morpholinoethanesulfonic acid buffer (MES 50 mM, [pH 5.7]) covered with a tap planchet (Art.611, Wohl- wend GmbH, Sennwald, Switzerland) and directly high pressure freezed using a High Pressure Freezing Machine HPF Compact 02 (Wohlwend GmbH, Sennwald, Switzerland). The samples were then chemically fixed, dehydrated and infiltrated with resin at cold temperature using the Leica AFS2 freeze substitution machine (Leica Mikrosysteme GmbH, Vienna, Austria) with the following protocol: Dehydration and fixation in a solution containing a mixture of osmium tetroxide 0.5% (EMS, Hatfield, PA) with glutaraldehyde 0.5% (EMS, Hatfield, PA) with uranyl acetate 0.1% (Sigma, St. Louis, MO) in acetone (Sigma, St Louis, MO, US) at graded temperature (−90 °C for 30 h; from −90 °C to −60 °C in 6 h; −60 °C for 10 h; from −60 °C to −30 °C in 6 h; −30 °C for 10 h; from −30 °C to 0° in 6 h) This was followed by washing in acetone and then infiltration in Spurr resin (EMS, Hatfield, PA, US) at graded concentration and temperature (30% for 10 h from 0 °C to 20 °C; 66% for 10 h at 20 °C; 100% twice for 10 h at 20 °C) and finally polymerized for 48 h at 60 °C in an oven.

### TEM tomography and 3D reconstruction

For electron tomography, semi-thin sections of 250 nm thickness were cut transversally to the root using a Leica Ultracut (Leica Mikrosysteme GmbH, Vienna, Austria) and then, picked up on 75 square mesh copper grids (EMS, Hatfield, PA, US). Sections were post-stained on both sides with uranyl acetate (Sigma, St Louis, MO, US) 2% in H_2_O for 10 min and rinsed several times with H_2_O. Protein A Gold 10 nm beads (Aurion, Wageningen, The Netherlands) were applied as fiducials on both sides of the sections and the grids were placed on a dual axis tomography holder (Model 2040, Fischione Instruments). The area of interest was taken with a transmission electron microscope JEOL JEM-2100Plus (JEOL Ltd., Akishima, Tokyo, Japan) at an acceleration voltage of 200 kV with a TVIPS TemCamXF416 digital camera (TVIPS GmbH, Gauting, Germany) using the SerialEM software^54^. Micrographs were taken as single tilt series over a range of −60° to +60° using SerialEM at tilt angle increment of 1°. Tomogram reconstruction, segmentation and model visualization were done with IMOD software^53^.

### Lignin staining with permanganate potassium (KMnO_4_) using TEM

Visualization of lignin deposition around Casparian strip was done using permanganate potassium (KMnO_4_) staining^55^. The sections were post-stained using 1% of KMnO_4_ in H_2_O (Sigma, St Louis, MO, US) for 45 min and rinsed several times with H_2_O. Micrographs were taken with a transmission electron microscope FEI CM100 (FEI, Eindhoven, The Netherlands) at an acceleration voltage of 80 kV with a TVIPS TemCamF416 digital camera (TVIPS GmbH, Gauting, Germany) using the software EM-MENU 4.0 (TVIPS GmbH, Gauting, Germany). Panoramic were aligned with the software IMOD.

### Detection of H_2_O_2_ production in situ using transmission electron microscopy

Visualization of H_2_O_2_ production around Casparian strip and *caspQ* dots was done by cerium chloride method^7,26^. Five‐day‐old *Arabidopsis* seedlings were incubated in 50 mM MOPS pH7.2 containing 10 mM CeCl_3_ for 30 min. After incubation with CeCl_3_, seedlings were washed twice in MOPS buffer for 5 min and fixed in glutaraldehyde solution (EMS, Hatfield, PA) 2.5% in 100 mM phosphate buffer (pH 7.4) for 1 h at room temperature. Then, they were post‐fixed in osmium tetroxide 1% (EMS) with 1.5% of potassium ferrocyanide (Sigma, St. Louis, MO) in phosphate buffer for 1 h at room temperature. Following that, the plants were rinsed twice in distilled water and dehydrated in ethanol solution (Sigma) at gradient concentrations (30% 40 min; 50% 40 min; 70% 40 min; two times (100% 1 h). This was followed by infiltration in Spurr resin (EMS) at gradient concentrations [Spurr 33% in ethanol, 4 h; Spurr 66% in ethanol, 4 h; Spurr two times (100% 8 h)] and finally polymerized for 48 h at 60 °C in an oven. Ultrathin sections 50 nm thick were cut transversally at 1.8 ± 0.1 mm from the root tip, on a Leica Ultracut (Leica Microsystems GmbH, Vienna, Austria) and picked up on a copper slot grid 2 × 1 mm (EMS) coated with a polystyrene film (Sigma). Micrographs were taken with a transmission electron microscope FEI CM100 (FEI, Eindhoven, The Netherlands) at an acceleration voltage of 80 kV with a TVIPS TemCamF416 digital camera (TVIPS GmbH, Gauting, Germany) using the software EM‐MENU 4.0 (TVIPS GmbH, Gauting, Germany).

### Biotin labelling, protein extraction and western blotting

Biotin labelling: For each sample, 160 mg of surface-sterilized seeds were imbibed in 0.2% agar and sown in described media containing mesh using 1 mL pipette along two lines per square plate. Seedlings were grown for 6 days at 22 °C continuous light, before transferring to 50 µM biotin containing half-strength MS media, under sterile conditions using the mesh to transfer whole seedling population of each plate. Seedlings were grown for another 24 h in biotin containing plates at 28 °C. One biological replicate, from each genotyped, comprised 10 plates.

*Protein Extraction:* Roots (100–120 mg per plate) were collected with razor blade, weighted and immediately frozen in 2 mL eppis containing a metal bead in liquid nitrogen. Per biological replicate from 10 plates, yielded app. 1 g of root material. Roots were ground using Tissue Lyzer II (Qiagen) with 2 cycles, 30 s, maximum speed, taking care to maintain material frozen, using pre-cooled racks at −80 °C and freezing samples in liquid-N_2_ between cycles. Protein extraction and biotinylated-protein purification was done adapting available protocols^31^ as follows: Ground roots were resuspended with Volume (2× weight) of RIPA buffer containing protease inhibitors (50 mM Tris, 150 mM NaCl, 0.1% (wt/vol) SDS, 0.5% (wt/vol) sodium deoxycholate and 1% (vol/vol) Triton X-100 in Millipore water, adjusted pH to 7.5 with HCl, 1 mM PMSF and 2.5× PIC-Complete (Roche) in cold by short vortexing and spin down at 6000 g for 10 min at 4 °C. For checking success of biotin feeding by western blot (*input*), 10 µL of extract were boiled in 4× Laemmli buffer supplemented with 20 mM DTT and 2 mM biotin at 95 °C for 5 min, and western blots performed as described below.

Supernatants were collected and dialyzed to remove free biotin with PD-10 columns (GE Healthcare) following manufacturer’s instructions. Dialyzed extracts were then incubated with 200 µL Dynabeads MyOne Streptavidin C1 (Invitrogen) overnight. Next day, beads were separated from protein extract using a magnetic rack, and washed with following sequence of buffers: 2× cold RIPA buffer, 1× cold KCl 1 M, 1× with cold, 1× cold 100 mM Na_2_CO_3_, 1× room temperature 2 M Urea in 10 mM Tris pH 8, and 2× cold RIPA buffer. For checking success of purification, 2% of the beads were boiled in 50 ml 4× Laemmli buffer supplemented with 20 mM DTT and 2 mM biotin at 95 °C for 5 min for immunoblots (*purified*). The rest of the beads was spun down to remove the remaining wash buffer, added 1× PBS and stored at −80 °C until further processing.

Western Blots of *input* and *purified* samples, proteins were separated in 4–12% gradient SDS-PAGE (Thermo scientific), and semi-dry transferred to Nitrocellulose membranes (Thermo scientific Pierce G2). For anti-GFP, membranes were blocked in PBS 0.5% Tween-20 5% Milk overnight, incubated 1 h in anti-GFP (rabbit polyclonal, 1:3000, Invitrogen), washed in 3 × 10 min PBS Tween 0.5%, incubated in anti-rabbit-HRP (1:5000, Promega) and washed in 3× 10 min PBS Tween 0.5% before detection. For streptavidin-HRP, membranes were blocked in TBS 0.1% Tween-20 5% Milk overnight, incubated 1 h in streptavidin-HRP (1:2000, Thermo Scientific), washed in 3 × 10 min TBS 0.1% Tween-20, 1 × 10 min TBS and 1 ×10 min destilled water before detection. Chemiluminescence was detected using SuperSignal West Pico kit (Thermo Scientific).

### Mass-spectrometry

Sample preparation: Protein digestion protocol on beads was adapted from^56^ (Han et al., 2017). Magnetic beads were washed with 500 ul of 2 M urea/50 mM TEAB (pH 7.8) and resuspended in 100 ul of the same solution supplemented with 1 mM DTT and 0.5 ug trypsin. After 1 h of incubation at 25 °C, the supernatant was collected, beads washed with 100 ul of 2 M urea/50 mM TEAB and this second supernatant pooled with the first one. After addition of 4 mM DTT (final concentration), the samples were reduced 30 min at 25 °C, and then alkylated with 20 mM chloroacetamide (final concentration) during 45 min at 25 °C in the dark. After addition of 0.5 ug of trypsin, the samples were digested overnight at 25 °C. The solutions were then acidified with 10 ul of 20% trifluoroacetic acid, desalted on a Sep-Pak tC18 Waters plate and eluted with 150 ul of 80% acetonitrile/0.1% formic acid.

Mass spectrometry analyses: Tryptic peptides fractions were dried and resuspended in 0.05% trifluoroacetic acid, 2% (v/v) acetonitrile, for mass spectrometry analyses. Tryptic peptide mixtures were injected on an Ultimate RSLC 3000 nanoHPLC system (Dionex, Sunnyvale, CA, USA) interfaced to an Orbitrap Fusion Tribrid mass spectrometer (Thermo Scientific, Bremen, Germany). Peptides were loaded onto a trapping microcolumn Acclaim PepMap100 C18 (20 mm × 100 μm ID, 5 μm, 100 Å, Thermo Scientific) before separation on a reversed-phase custom packed nanocolumn (75 μm ID × 40 cm, 1.8 μm particles, Reprosil Pur, Dr. Maisch). A flowrate of 0.25 μl/min was used with a gradient from 4 to 76% acetonitrile in 0.1% formic acid (total time: 140 min). Full survey scans were performed at a 120,000 resolution, and a top speed precursor selection strategy was applied to maximize acquisition of peptide tandem MS spectra with a maximum cycle time of 0.6 s. HCD fragmentation mode was used at a normalized collision energy of 32%, with a precursor isolation window of 1.6 m/z, and MS/MS spectra were acquired in the ion trap. Peptides selected for MS/MS were excluded from further fragmentation during 60 s.

Data analysis: Tandem MS data were processed by the MaxQuant software (version 1.6.3.4)^57^ (Cox and Mann, 2008) incorporating the Andromeda search engine^58^ (Cox et al., 2011). An *Arabidopsis thaliana* reference proteome database from UniProt (November 2019 version, 39,362 sequences), supplemented with sequences of common contaminants, was used. Trypsin (cleavage at K,R) was specified as the enzyme definition, allowing 2 missed cleavages. Carbamidomethylation of cysteine was specified as a fixed modification, N-terminal acetylation of protein and oxidation of methionine as variable modifications. All identifications were filtered at 1% FDR at both the peptide and protein levels with default MaxQuant parameters. Protein abundances were calculated using iBAQ, ‘intensity-based absolute quantification’, i.e., protein intensities (the sum of all identified peptide intensities) divided by the number of theoretically observable peptides (calculated by in silico protein digestion), and values were log2-transformed for visualization purposes^59^. MaxQuant data were further processed with Perseus software (version 1.6.14.0)^60^ for the filtering, log2-transformation and normalization of values, data imputation, statistical analyses and GO annotations. The mass spectrometry proteomics data have been deposited to the ProteomeXchange Consortium via the PRIDE partner repository, with the number PXD035124. GO annotation enrichment test was performed based on^61^. Sub-cellular localizations were queried in SUBA4 database^62^, following consensus algorithm.

### Reporting summary

Further information on research design is available in the Nature Portfolio Reporting Summary linked to this article.

## Supplementary information

Supplementary Information

Description of Additional Supplementary Files

Supplementary Data 1

Supplementary Data 2

Supplementary Movie 1

Supplementary Movie 2

Reporting Summary

## Data Availability

Genetic constructs, transgenic lines and imaging data generated in the course of this study are available from the corresponding author on request. The mass spectrometry proteomics data have been deposited to the ProteomeXchange Consortium via the PRIDE partner repository with the dataset identifier PXD035124 PXD035124. Source data are provided with this paper Source data are provided with this paper.

## Source data

Source Data file
