# Supplementary material for: Directed growth and fusion of membrane-wall microdomains requires CASP-mediated inhibition and displacement of secretory foci
Source: Nat Commun. 2023 Mar 23;14:1626. doi: 10.1038/s41467-023-37265-7 (PMC10036488; doi:10.1038/s41467-023-37265-7)
Supplement: Supplementary file 1 — Supplementary Information [file 41467_2023_37265_MOESM1_ESM.pdf]

Supplementary Figure 1

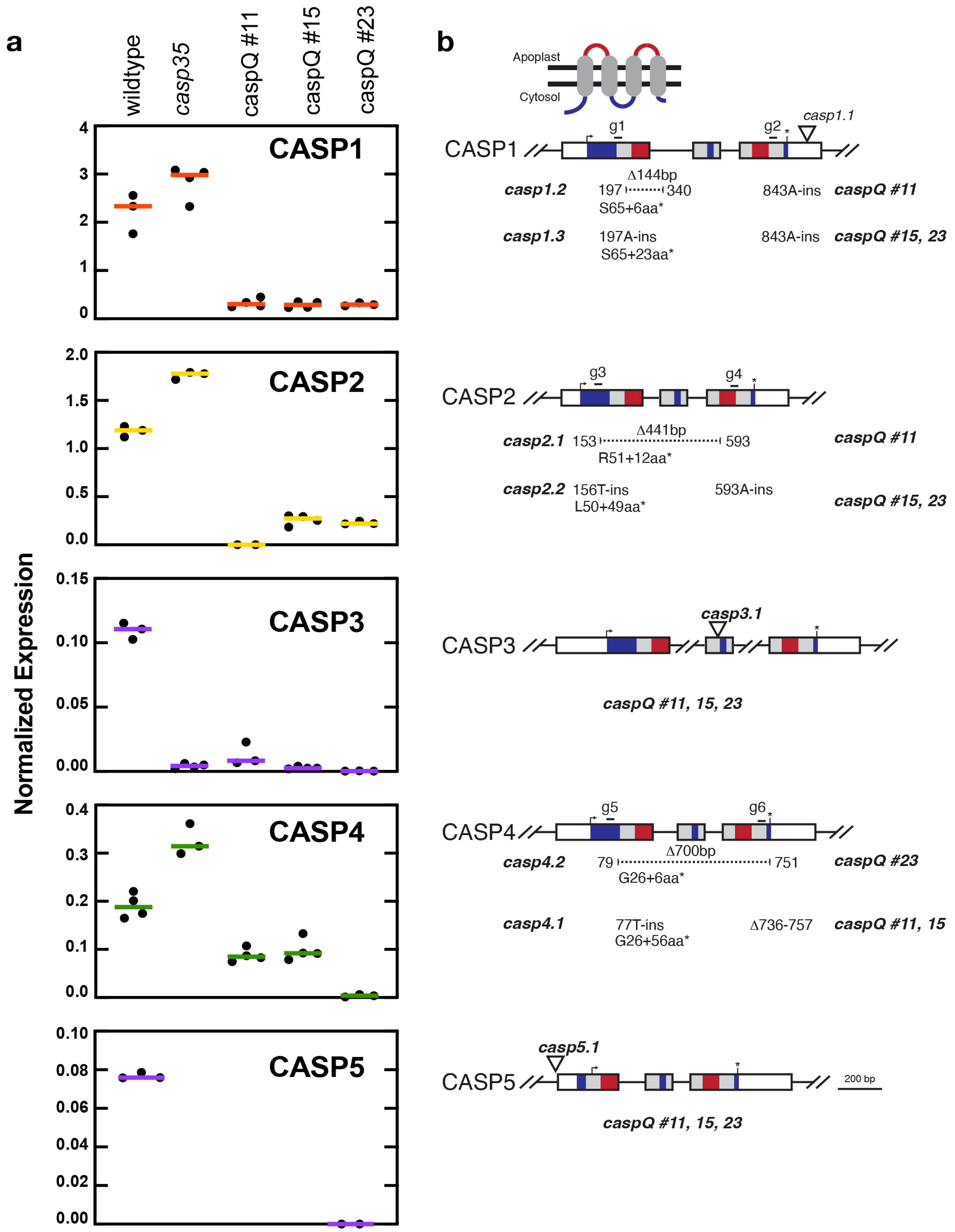

Supplementary Figure 1: CASP expression and CRISPR-Cas9 targeting strategy in caspQ

a. Normalized gene expression of *CASP1-CASP5* genes in wild-type, T-DNA mutant *casp35* and CRISPR-Cas9 *caspQ* alleles. Expression normalized to *Clathrin adaptor complexes medium subunit family protein* (AT4G24550). **b.** CRISPR-Cas9 gene targeting of *CASP1*, *CASP2* and *CASP4* and predicted early STOP codon alleles in *casp35* T-DNA mutant background. White boxes indicate 5′ and 3′UTR, coloured boxes predicted protein domains (blue, intracellular loops; gray, transmembrane domains; red, extracellular domains); top-right arrow, START; *, STOP codon; *g1-g6*, guide RNAs; Δ, deletions; ins, insertions; aa, amino-acid position.

Supplementary Figure 2

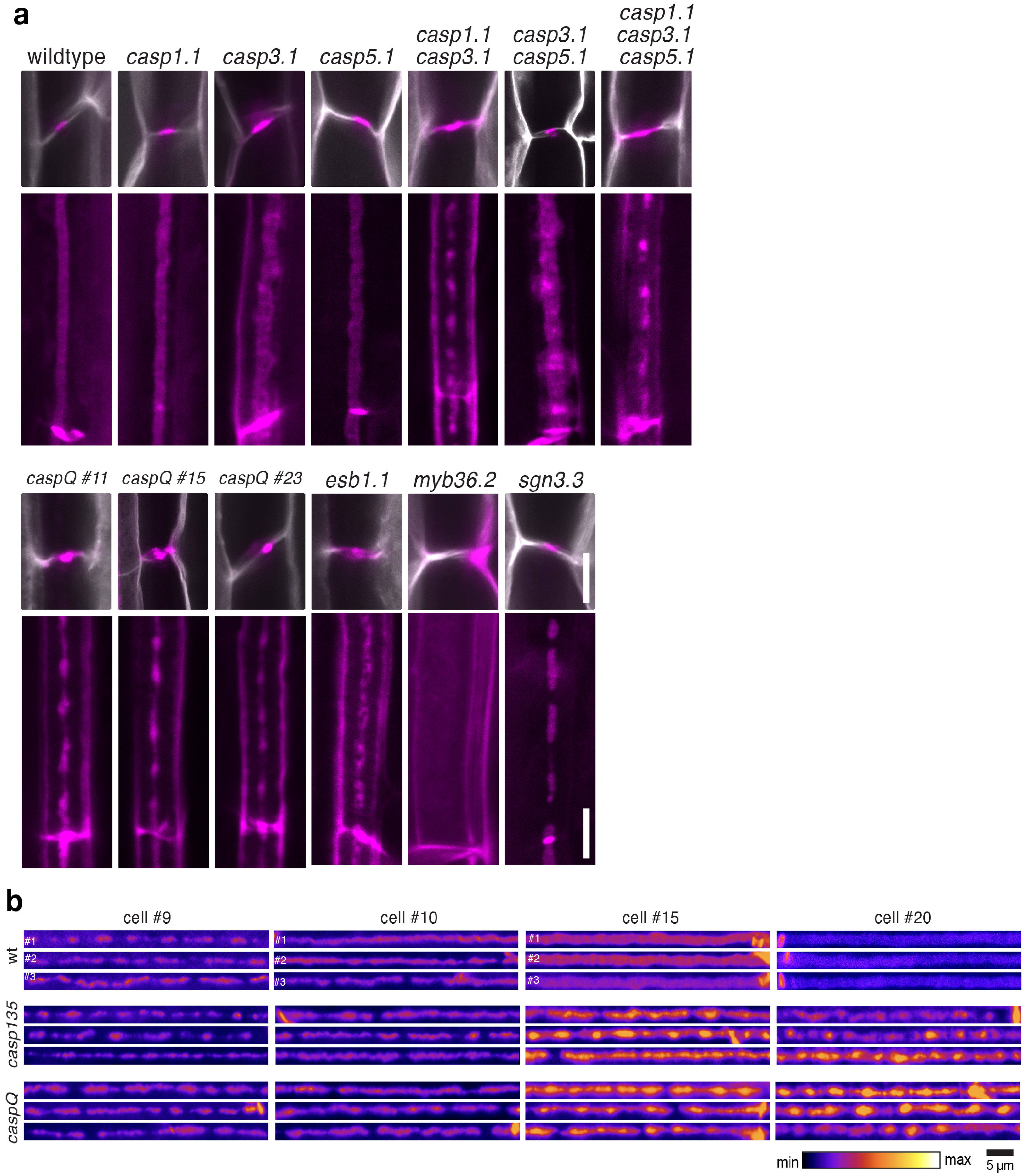

Supplementary Figure 2: Representative pictures of lignification in mid and surface views

a. Mid (upper) and surface (lower panel) views of lignin (magenta, Basic Fuchsin) and cell wall (gray, calcofluor white) staining at endodermal cell number 20 of additional mutants analysed in Figure 1. Pictures of same genotypes are identical to main figure. **b.** Representative pictures of CS surface view from three individuals at endodermal cell numbers 9, 10, 15 and 20 from wild-type, *casp135* and *caspQ*. Scale bars 5 µm.

Supplementary Figure 3

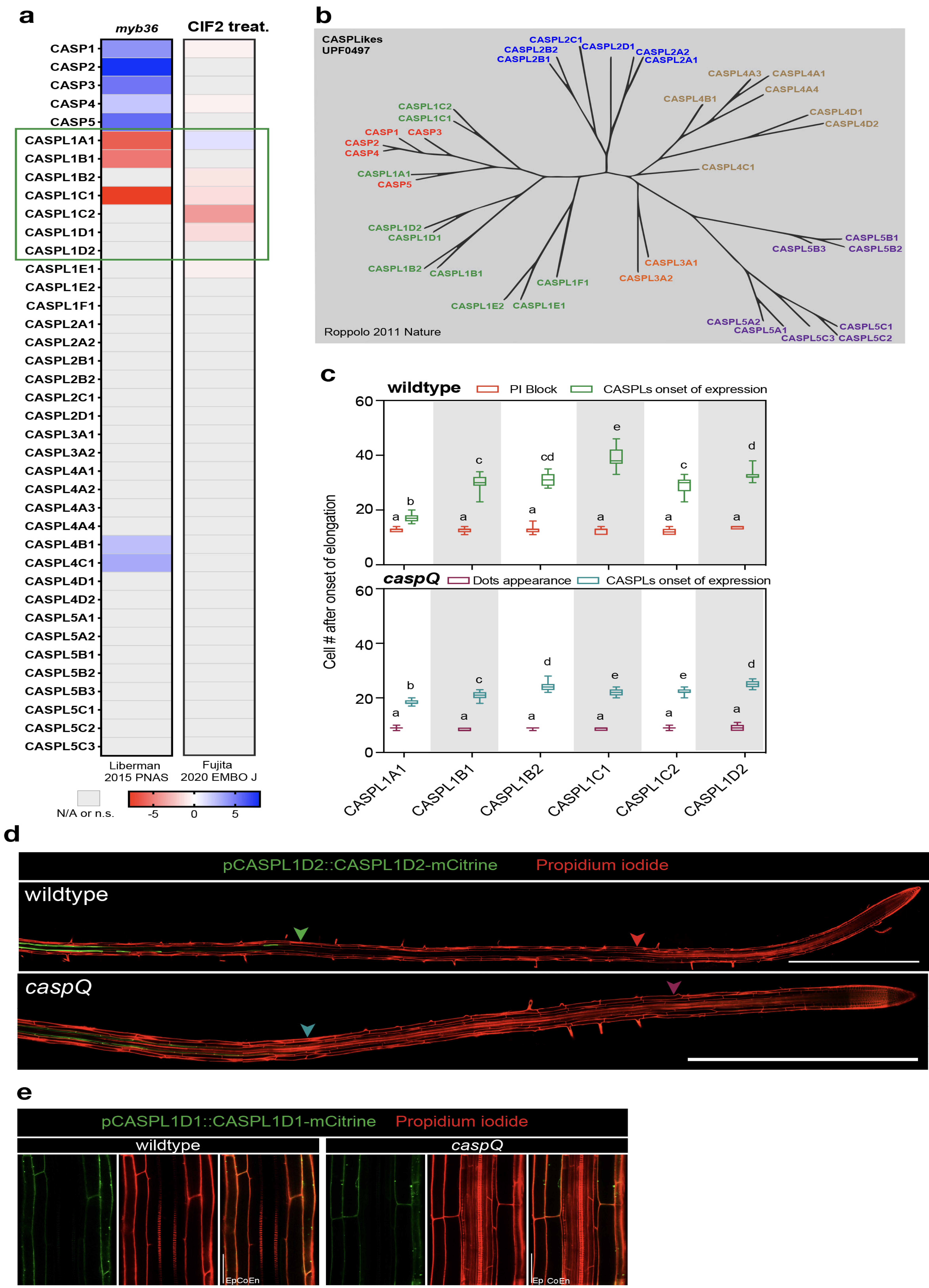

Supplementary Figure 3

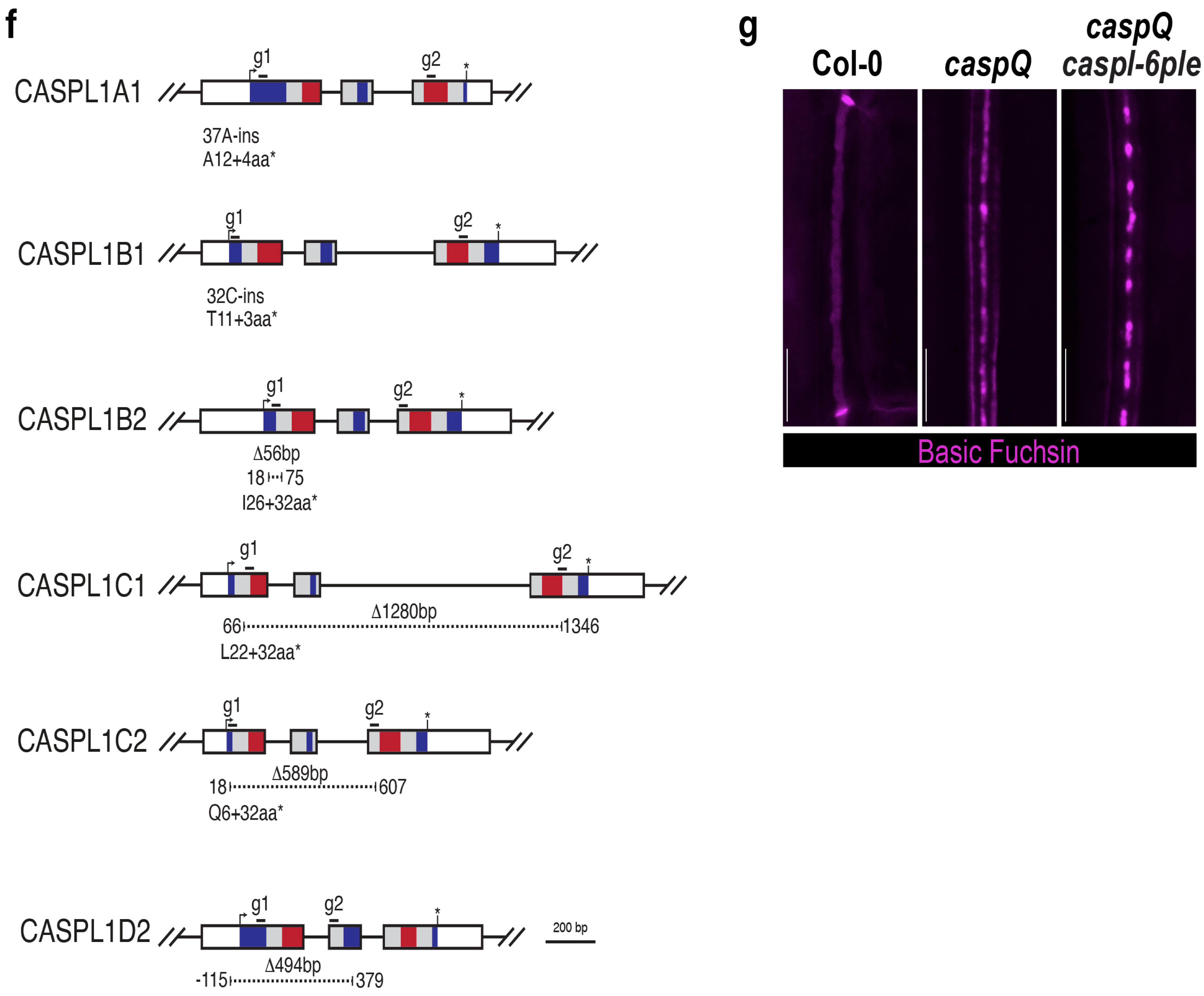

Supplementary Figure 3: Other CASP-LIKES are not involved in CS formation

a. Comparative expression data of CASP-LIKE genes in endodermal and/or CS-relevant RNA-seq experiments: *myb36* mutant differential expression (Liberman et al., 2015) and CIF2 treatment (Fujita et al., 2020) **b.** CASPL phylogenetic tree, adapted from (Roppolo et al., 2014). **c-d.** Analysis of spatial expression of translational fusions *pCASPLx::CASPLx-mCitrine* (*CASPL1A1, CASPL1B1, CASPL1B2, CASPL1C1, CASPL1C2* and *CASPL1D2*) in wild-type and *caspQ* using PI-block (in wild-type) or PI-stained foci (in *caspQ*) as reference for endodermal differentiation. **d.** Representative root pictures taken for analysis in (c) for *pCASPL1D2::CASPL1D2-mCitrine*. Scale bar 1 mm. **e.** Representative pictures for pCASPL1D1::-CASPL1D1-mCitrine at cell 20 after onset of elongation. Scale bar 50µm. **f.** CRISPR-Cas9 gene targeting of *CASPL1A1, CASPL1B1, CASPL1B2, CASPL1C1, CASPL1C2* and *CASPL1D2* in the *caspQ* background (*caspQ caspl-6ple*). White boxes 5′ and 3′UTR, coloured boxes predicted protein domains (blue, intracellular loops; gray, transmembrane domains; red, extracellular domains); top-right arrow, START; *, STOP codon; g1-g6, guide RNAs; Δ, deletions; ins, insertions; aa, amino-acid position. g. CS surface views stained with Basic Fuchsin (lignin, magenta) of wild-type, *caspQ* and *caspQ caspl-6ple*. Scale bar 10µm.

Supplementary Figure 4

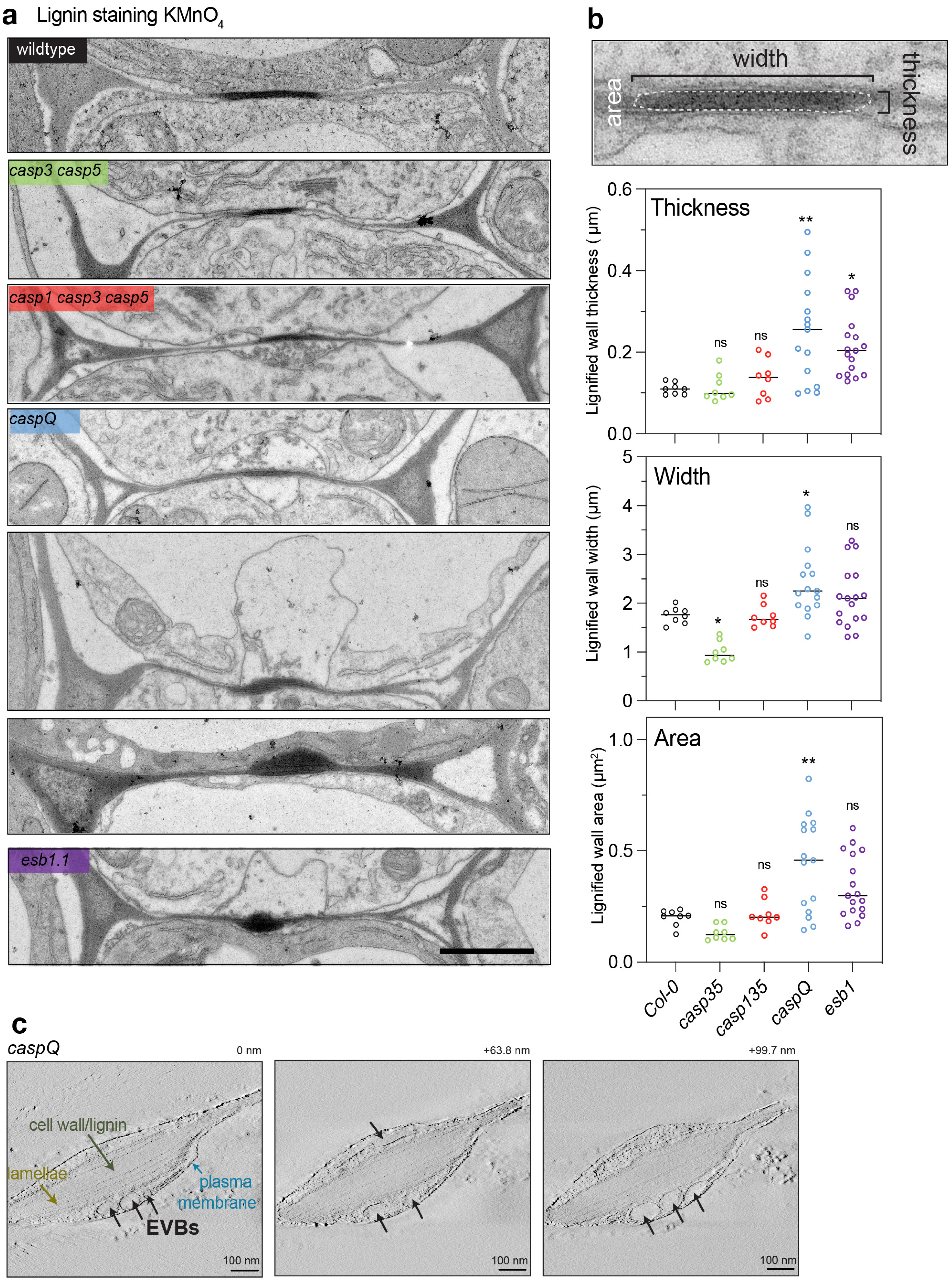

Supplementary Figure 4: Representative electron-micrographs of all genotypes analysed

a. Representative electron micrographs with $KMnO_4$ lignin staining of endodermal cell-cell contacts in transversal cuts at 1.8 mm from root tip from wild-type, *casp35, casp135, caspQ* and *esb1*. For *caspQ* three distinctive appearances are shown. **b.** Parameter quantification on central-aligned lignified walls stained by $KMnO_4$: thickness, width and area. Graphs show measurements of n~16 cell-cell contacts from 2 individuals; mean (line) and significant differences to wild-type by ANOVA, Tukey test p-value < 0.05. Scale bars 10 µm. **c.** Series of three optical sections from the tomogram of high-pressure freezing samples from a lignified foci of *caspQ* mutant. Scale bar 0.1 µm.

Supplementary Figure 5

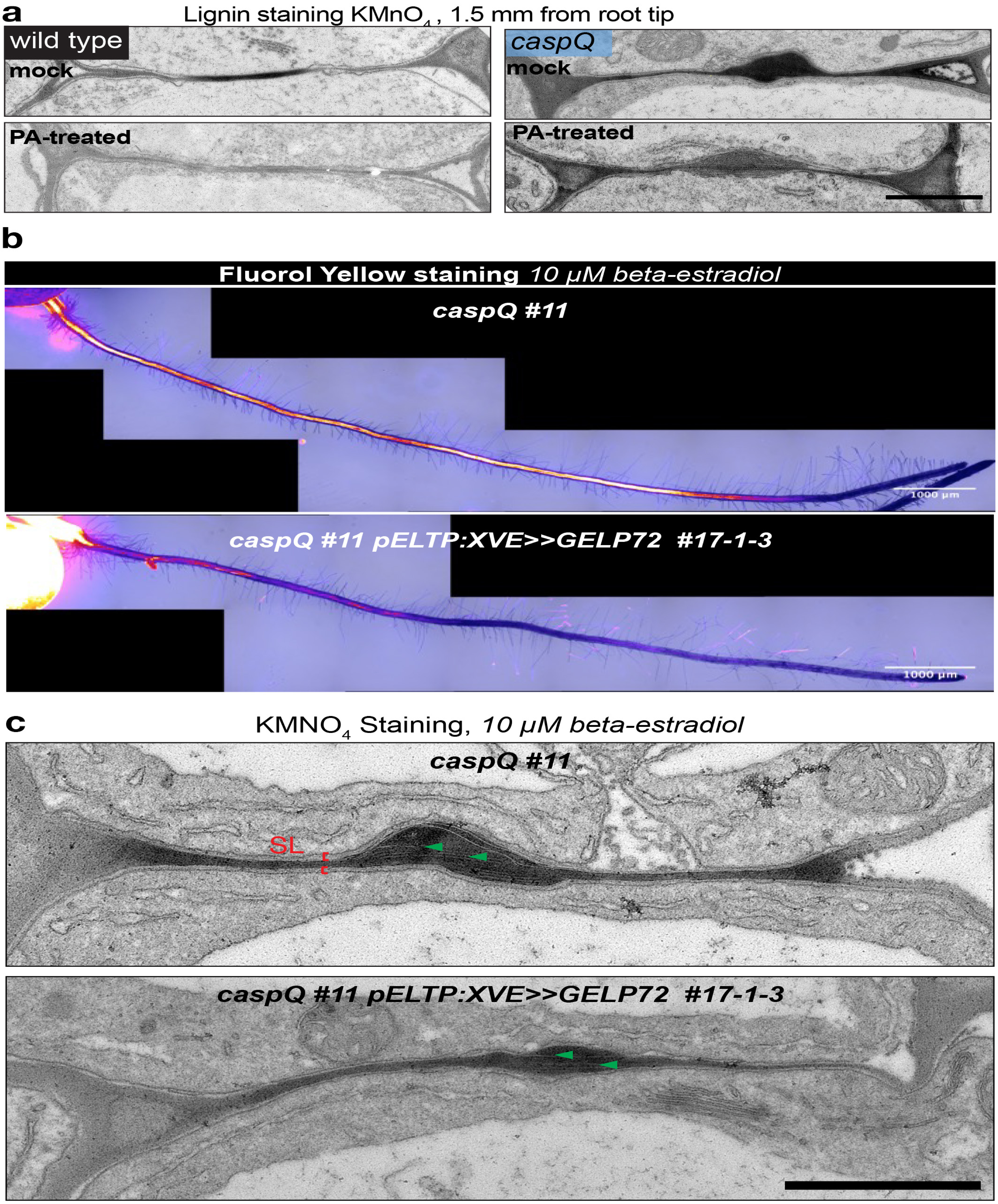

Supplementary Figure 5: caspQ microdomains are made of lignin and an unknown matrix

a. Representative electron micrographs with $KMnO_4$ lignin staining from wild-type and *caspQ* grown in mock or in presence of lignin biosynthesis inhibitor PA. Scale bars 1 µm.

b-c. Fluorol Yellow suberin staining **(b)** and Electron microgrpahs **(c)** of 5d old seedlings grown in 10µM β-estradiol of wild-type, *caspQ* and *caspQ* pELTP:XVE>>GELP72 (pRU182) #17-1-3. Scale bars 1 mm (b) and 1 µm (c).

Supplementary Figure 6

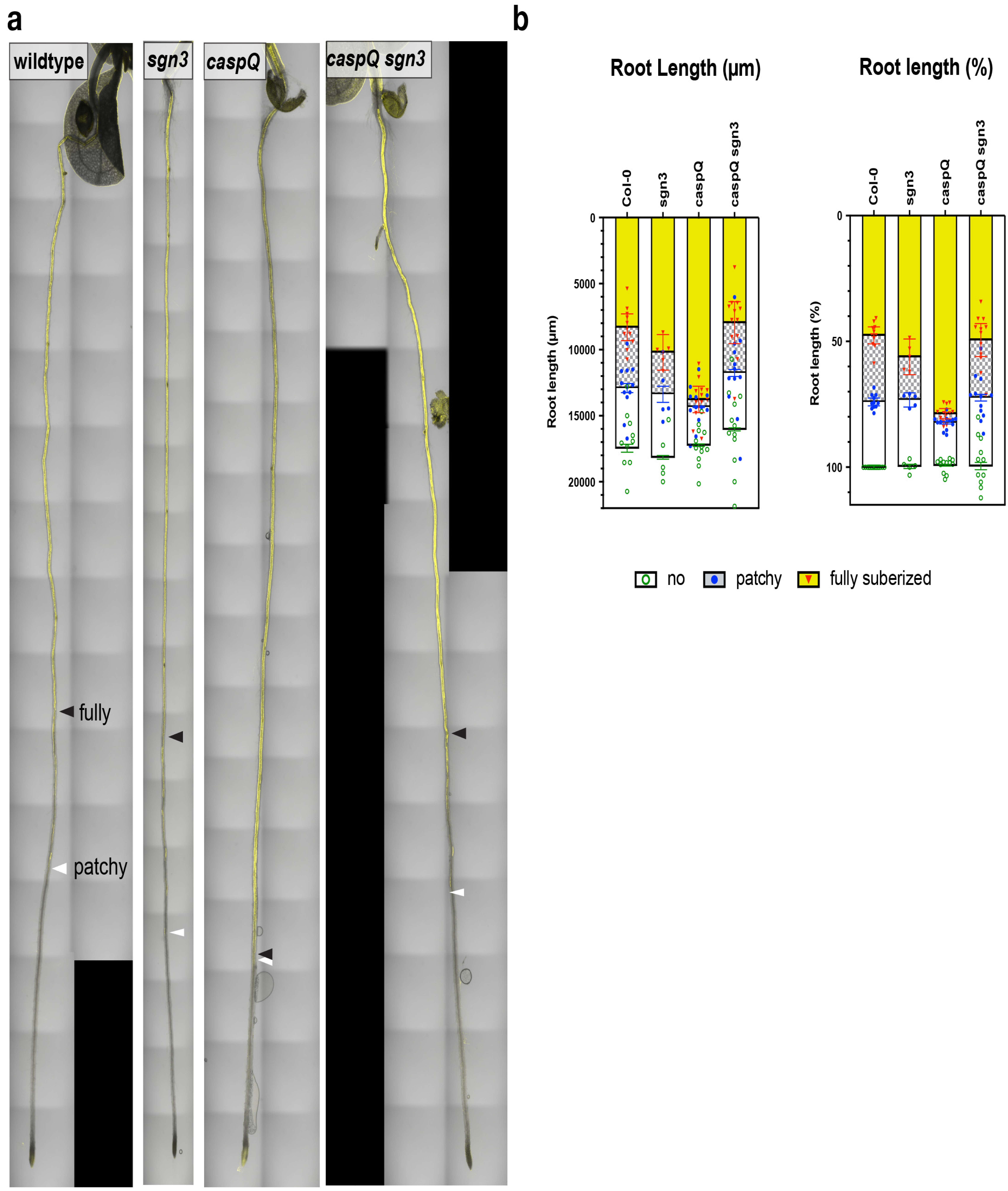

Supplementary Figure 3.1: caspQ ectopic suberin is SGN3-dependent

a-b. Fluorol Yellow suberin staining in *wild-type*, *sgn3*, *caspQ* and *caspQ sgn3* 5d old-seedlings. Confocal pictures **(a)** and quantification of endodermal suberin patterns **(b):** no suberin (white), patchy (gray) and fully suberized (yellow) as function of absolute (left) and relative (%, right) root length to *wild-type*.

Supplementary figure 7

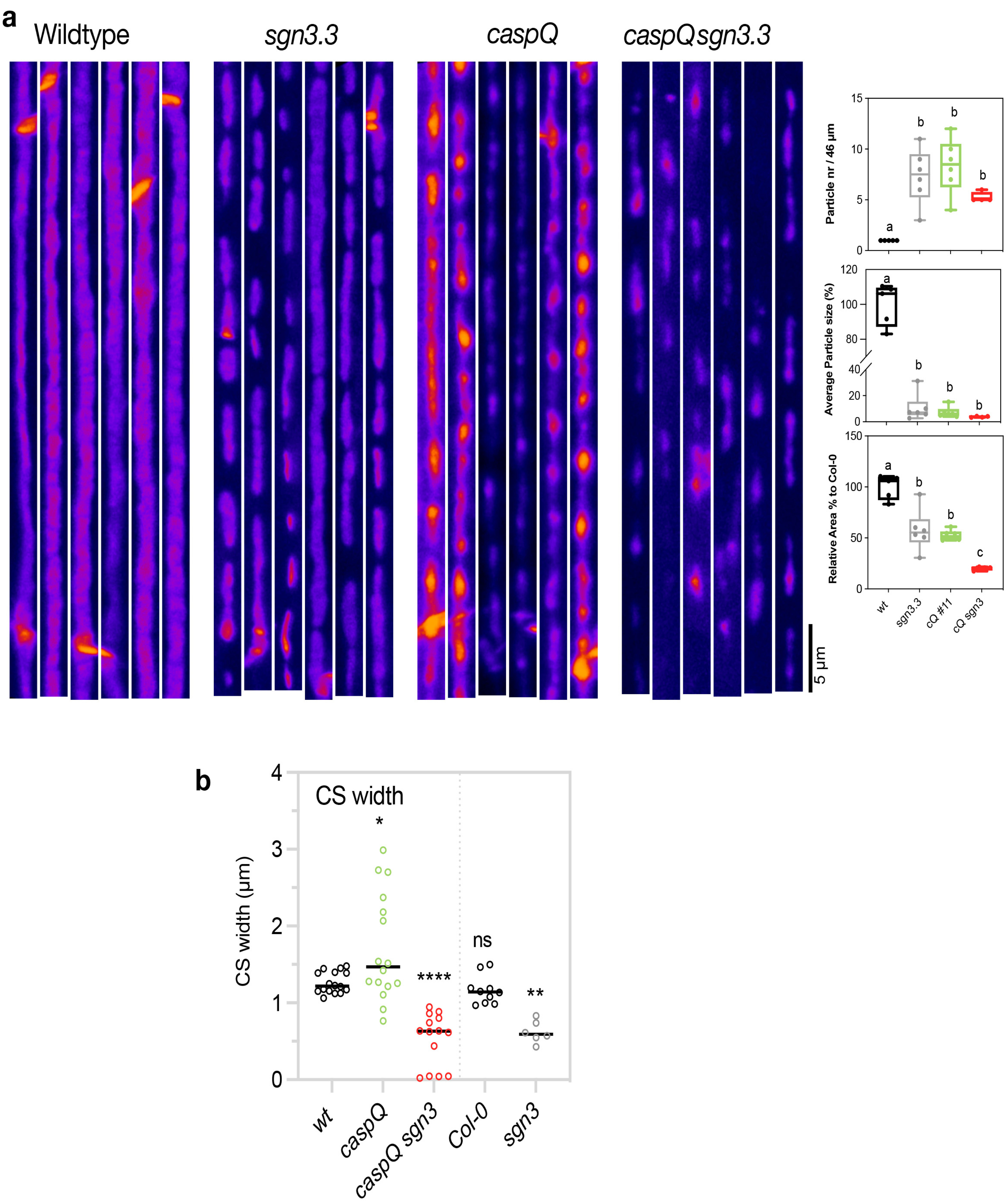

Supplementary Figure 7: caspQ sgn3 lignification is strongly reduced

a. Pictures of CS surfaces used for particle analysis FIJI-plugin and respective derived parameters from wild-type, *sgn3*, *caspQ* and *caspQ sgn3*, as shown in Fig. 3b. **b.** Quantification CS width from EM pictures of *wild-type*, *sgn3*, *caspQ* and *caspQ sgn3* as shown in Fig.3c-d. Scale bar 5 µm.

Supplementary figure 8

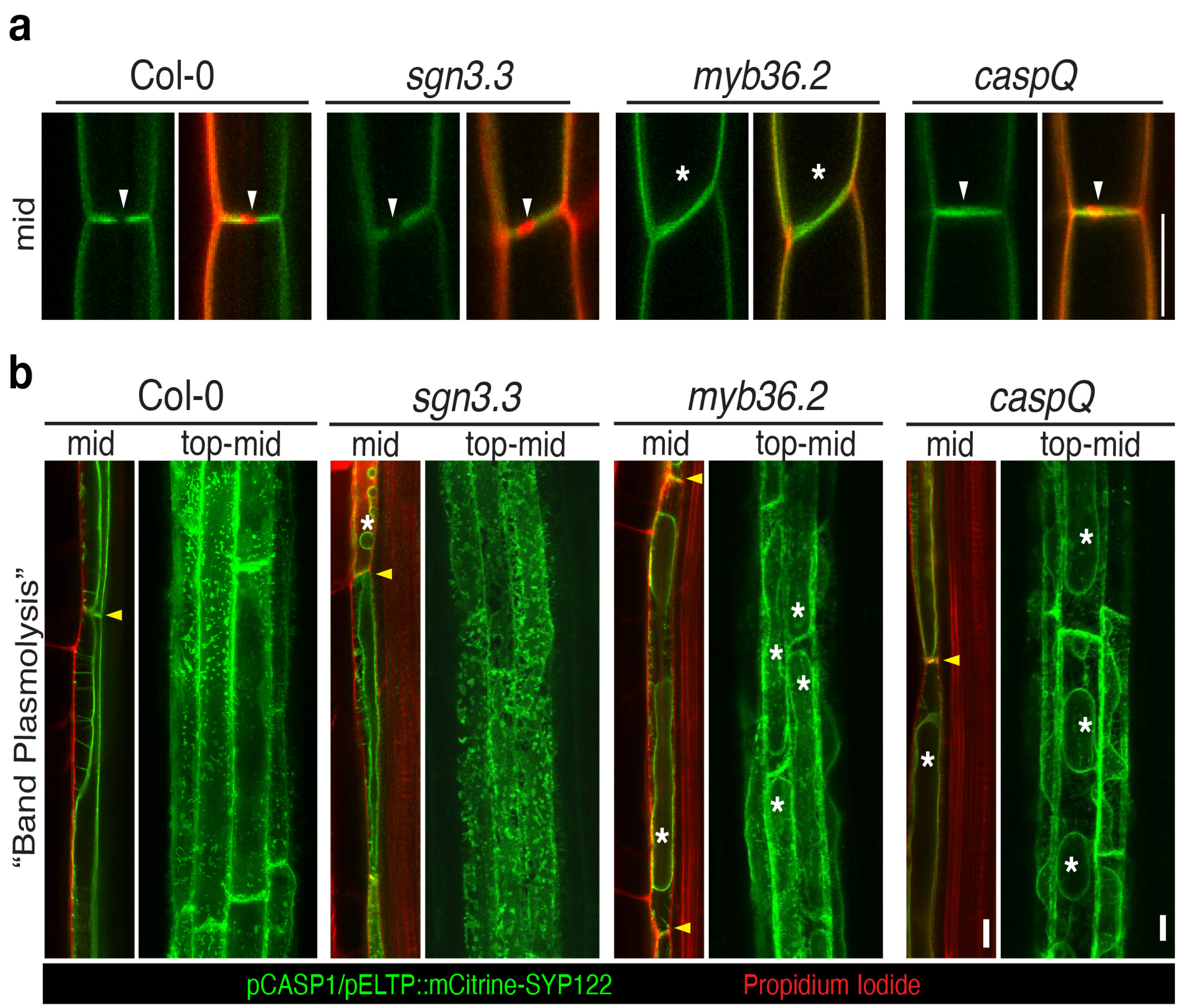

Supplementary Figure 8: CSD exclusion zone and plasma membrane wall attachment after plasmolysis in wild-type, sgn3, myb36 and caspQ

a. Plasma-membrane marker mCitrine-SYP122 is excluded from CSD labelled by the PI-stained CS in wild-type and *sgn3* (white arrows) but is freely diffused in transversal membranes of *myb36* and of *caspQ*, with difference that *myb36* lacks a CS (asterisk) but *caspQ* still forms a CS (white arrow). **b.** Plasma membrane marker mCitrine-SYP122 and PI-stained cell-walls after plasmolysis in seedlings mounted in 0.8M Mannitol solution. For wild-type in mid-view band-plasmolysis can be observed (yellow arrow), and but no obvious detachment appears in top-mid view. In *sng3* mid-view protoplasts (*) but in top-mid view only mild detachments. *caspQ* and *myb36* display very similar behaviour, with clear protoplasts (*) in either mid or trop-mid views.

Supplementary Figure 9

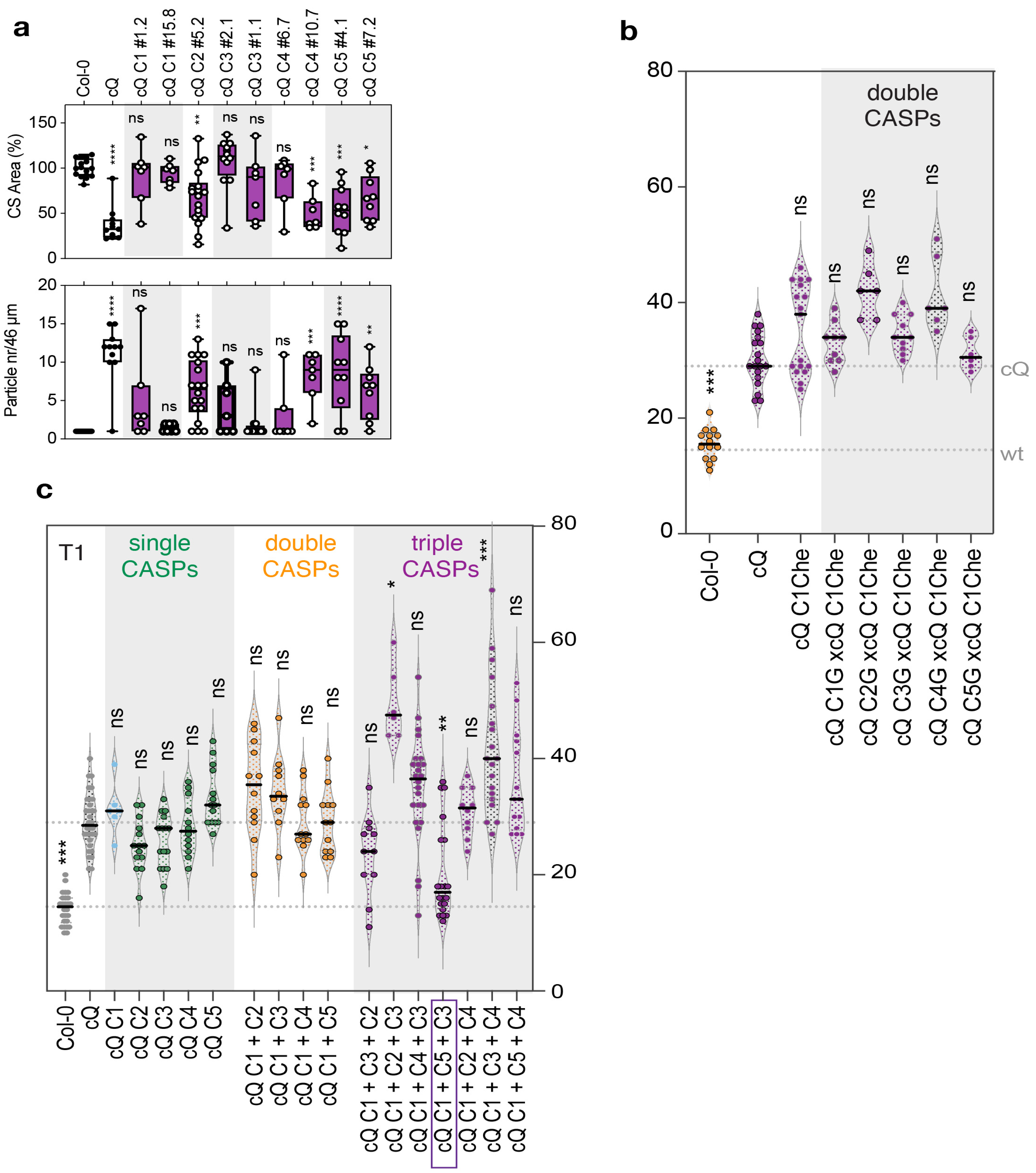

Supplementary Figure 9: Multiple CASP combinations reveals caspQ complementation requires at least three CASP genes.

a. Particle analysis in CS surface pictures as in (c) for wild-type, *caspQ* and complementation *caspQ* + pCASPn:CASP-GFP, n=8-12. Analysis done as in Fig. 1, yield CS Area (%) relative to wild-type (upper graph), and particle number along ~46 µm of CS length. Statistical differences by ANOVA, Tukey's test comparisons to wild-type (p<0.05). **b-c.** Propidium iodide uptake assay in *caspQ* complementation lines **(b):** double CASP combinations obtained by crossing *caspQ* + pCASP1:CASP1-mCherry with *caspQ* + pCASPn:CASPn-GFP. **(c):** single, double and triple CASP1-5 complementation of *caspQ* obtained by combinatorial gateway constructs with one, two or three CASPs in T1, n=12-15 individuals (b). Statistical differences

Supplementary Figure 10

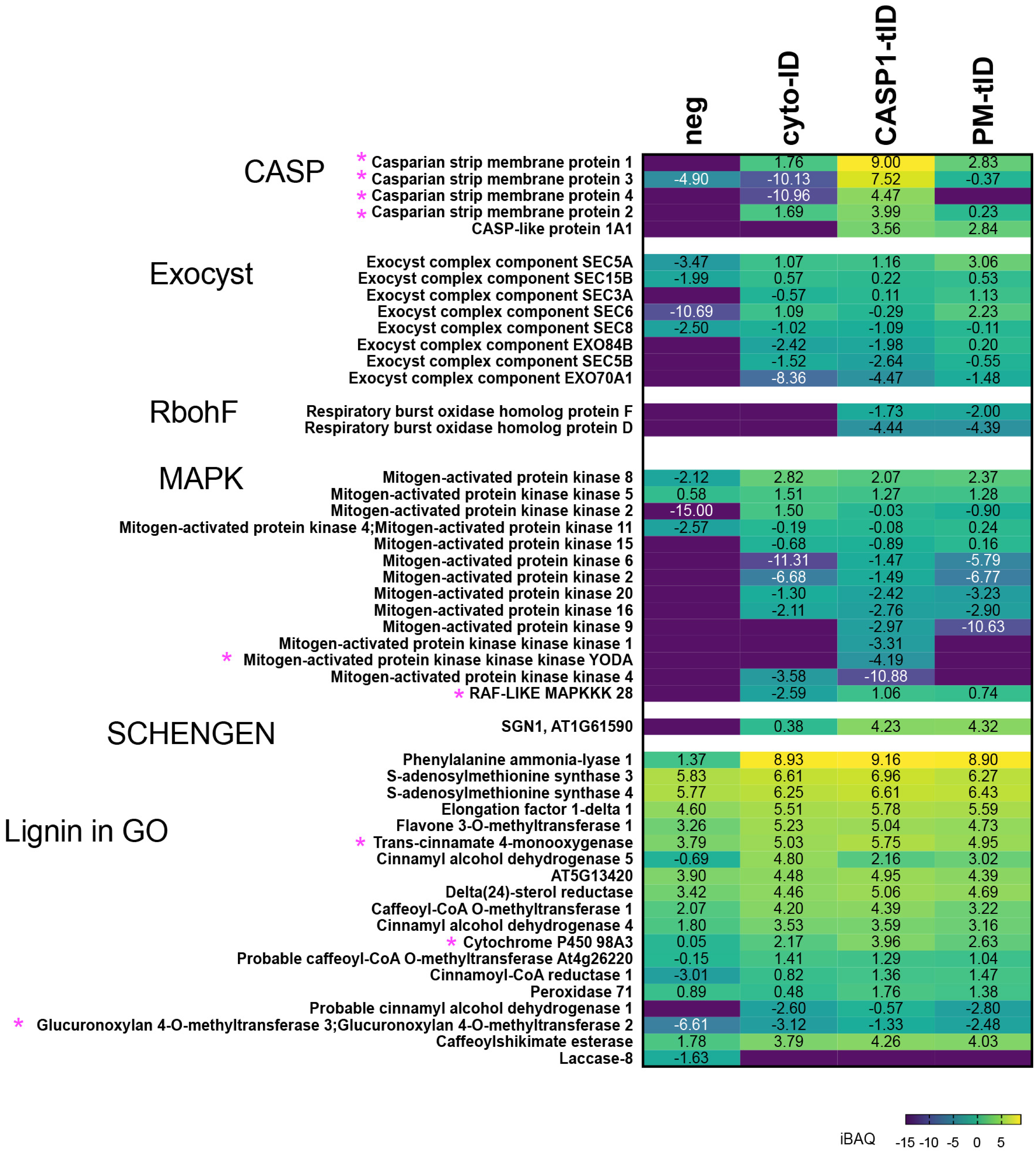

Supplementary Figure 10: Profile of CS associated proteins in Endodermal turboID proximity labelling experiment

Detected members, abundances in TurboID samples (log2 iBAQ) and their significant enrichment (p<0.05) in CASP1-tID sample (asterisk), from protein families or GO-terms expected to be involved in CS formation. *CASP-Like family*: 5 out of 39 members were identified in the experiment, four of which (CASP1 to CASP4) significantly enriched in CASP1-tID. *Exocyst*: one member of each subunit of exocyst complex was detected, with exceptions of subunit SEC10, for which no member was identified; *RBOH* (Respiratory Oxidative Burst Homologue): two isoforms involved in CS (RBOHF) and CS-surveillance endodermal lignification (RBOHD) (Fujita et al 2020); *Mitogen-Activated protein kinases:* detected members from MAPKKK, MAPKK and MPK families are shown; *SCHENGEN* pathway: among SGN1 and SGN3, only SGN1 was identified; *Lignin in GO-term:* We surveyed for the GO-term lignin, and could detect three CASP1-tID specific proteins, i.e. trans-cinnamate 4-monoxygenase (or C4H, Cytochrome P450 73), cytochrome P450 98A3 and Glucuronoxylan 4-O-methyltransferase 3 and/or 2, which are predicted to localize to ER and/or Golgi membranes, according to SUBA4 (see Supplementary Fig. 11). *neg*, negative control, pCASP1::CASP1-GFP; *cytoID*, pCASP1::GFP-turboID; *CASP1tID*, pCASP1::CASP1-GFP-turboID, and *PM-tID*, pCASP1::GFP-turboID-SYP122.

Supplementary Figure 11

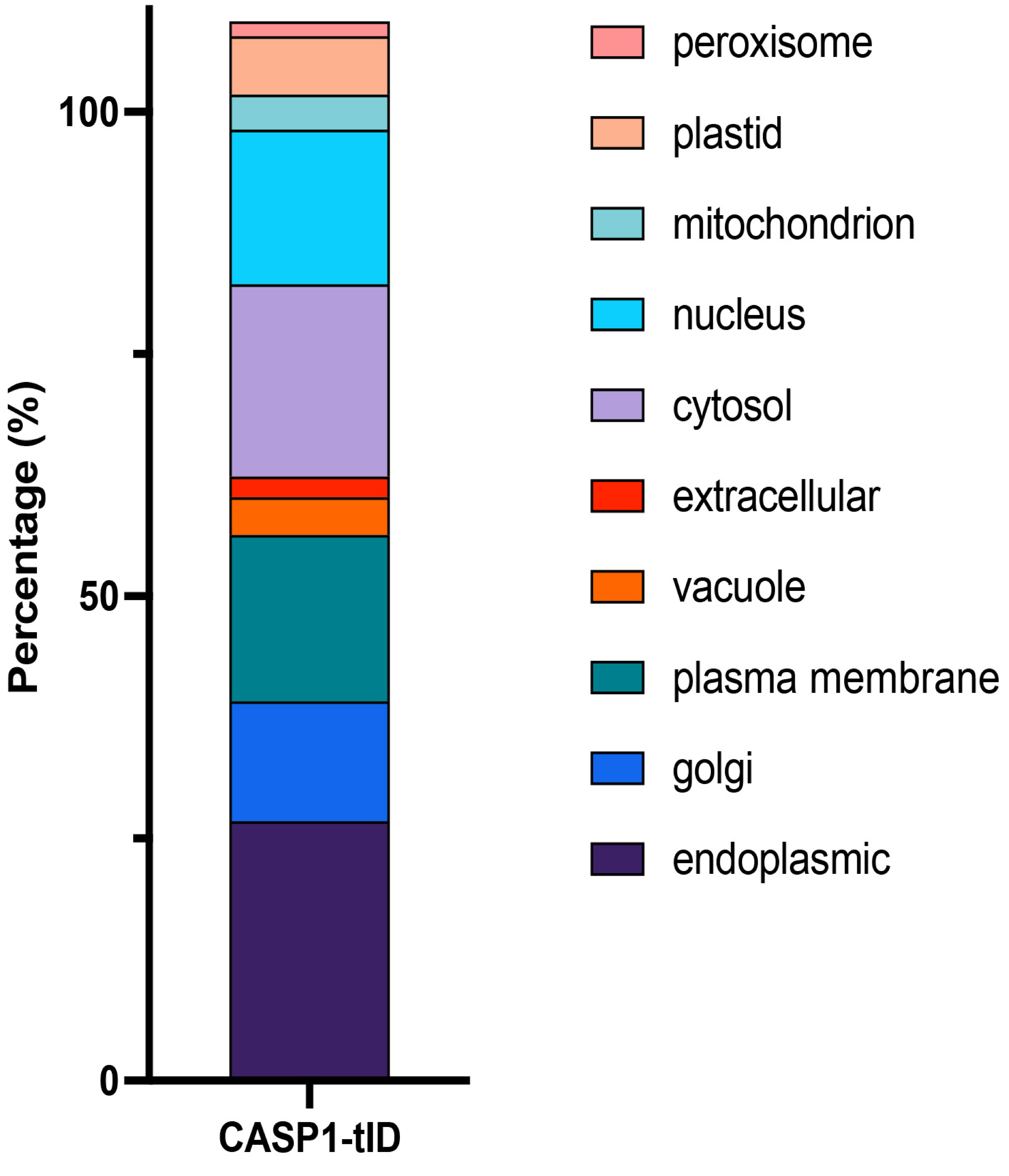

Supplementary Figure 11: Predicted sub-cellular localization of CASP1-proximal proteins

a. Relative (%) sub-cellular localizations of 332 CASP1-tID enriched proteins as predicted by SUBA4, consensus method. Note many proteins have dual/multiple localizations, thus sum of % is above 100%.

Supplementary Figure 12

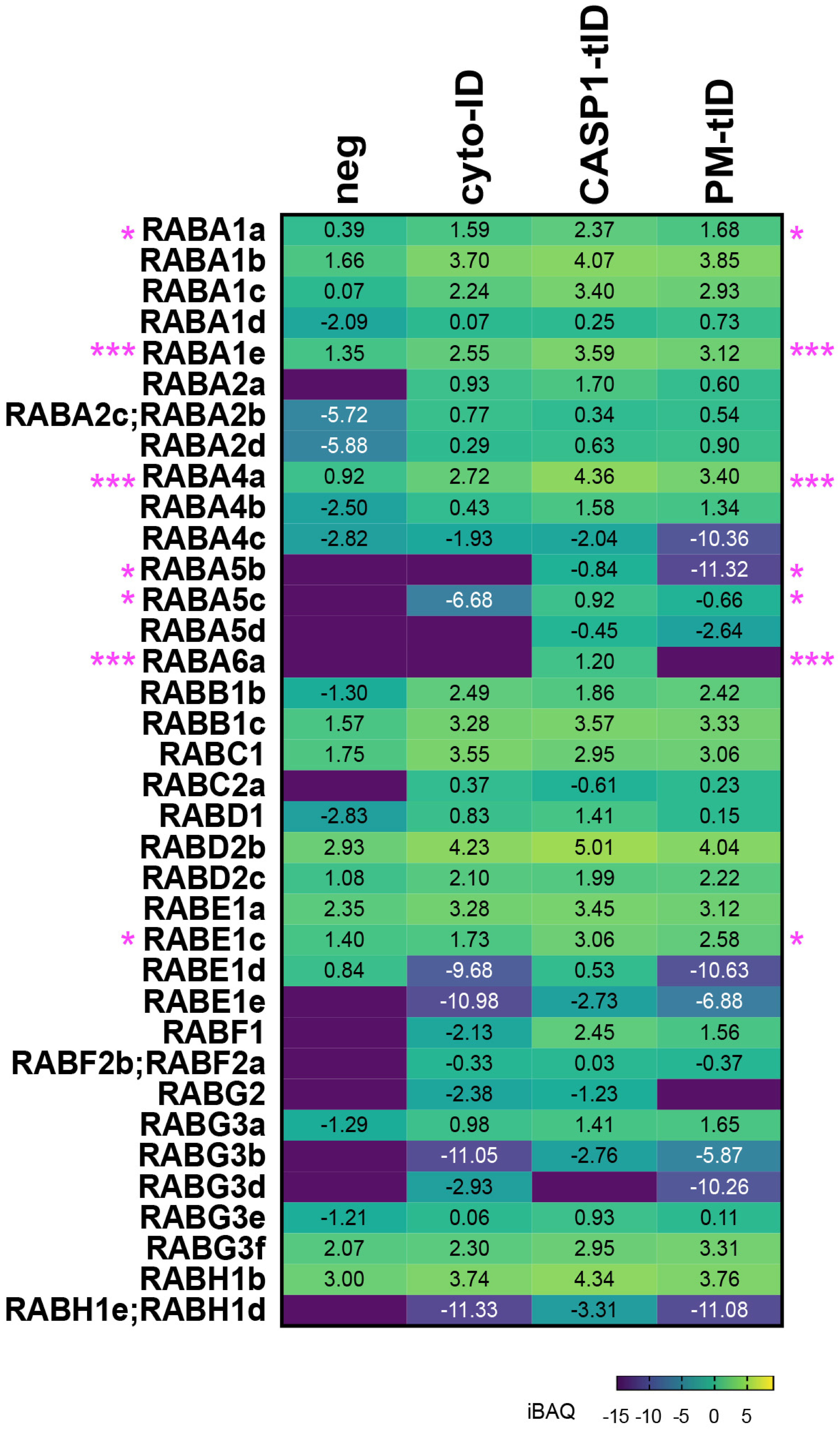

Supplementary Figure 12: Profile of Rab-GTPase family in Endodermal turboID proximity labelling experiment

Detected members from Rab-GTPase family and respective protein abundances in TurboID samples (log2 iBAQ). Significant enrichment ($p<0.05$, * and $p<0.01$, ***) in CASP1-tID sample.
