# Supplementary material for: Directed growth and fusion of membrane-wall microdomains requires CASP-mediated inhibition and displacement of secretory foci
Source: Nat Commun. 2023 Mar 23;14:1626. doi: 10.1038/s41467-023-37265-7 (PMC10036488; doi:10.1038/s41467-023-37265-7)
Supplement: Supplementary file 2 — Description of Additional Supplementary Files [file 41467_2023_37265_MOESM2_ESM.pdf]

Description of Additional Supplementary Files:

Supplementary Data 1: List of plant materials , plasmids and primers List of materials used in this study.

Supplementary Data 2: LC-MS/MS identified proteins in endodermal TurboID experiment

Supplementary Movie 1: Tomogram of caspQ microdomains from chemically fixed seedlings

Supplementary Movie 2: Tomogram of caspQ microdomains from high-pressure freezing fixed seedlings
